# Gis1 and Rph1 Regulate Glycerol and Acetate Metabolism in Glucose Depleted Yeast Cells

**DOI:** 10.1371/journal.pone.0031577

**Published:** 2012-02-21

**Authors:** Jakub Orzechowski Westholm, Susanna Tronnersjö, Niklas Nordberg, Ida Olsson, Jan Komorowski, Hans Ronne

**Affiliations:** 1 Department of Medical Biochemistry and Microbiology, Uppsala University, Uppsala, Sweden; 2 Linnaeus Centre for Bioinformatics, Uppsala University, Uppsala, Sweden; 3 Department of Plant Biology and Forest Genetics, Swedish University of Agricultural Sciences, Uppsala, Sweden; 4 Department of Microbiology, Swedish University of Agricultural Sciences, Uppsala, Sweden; 5 Interdisciplinary Centre for Mathematical and Computational Modelling, University of Warsaw, Warsaw, Poland; 6 Department of Cell and Molecular Biology, Science for Life Laboratory, Uppsala University, Uppsala, Sweden; University of Minnesota, United States of America

## Abstract

Aging in organisms as diverse as yeast, nematodes, and mammals is delayed by caloric restriction, an effect mediated by the nutrient sensing TOR, RAS/cAMP, and AKT/Sch9 pathways. The transcription factor Gis1 functions downstream of these pathways in extending the lifespan of nutrient restricted yeast cells, but the mechanisms involved are still poorly understood. We have used gene expression microarrays to study the targets of Gis1 and the related protein Rph1 in different growth phases. Our results show that Gis1 and Rph1 act both as repressors and activators, on overlapping sets of genes as well as on distinct targets. Interestingly, both the activities and the target specificities of Gis1 and Rph1 depend on the growth phase. Thus, both proteins are associated with repression during exponential growth, targeting genes with STRE or PDS motifs in their promoters. After the diauxic shift, both become involved in activation, with Gis1 acting primarily on genes with PDS motifs, and Rph1 on genes with STRE motifs. Significantly, Gis1 and Rph1 control a number of genes involved in acetate and glycerol formation, metabolites that have been implicated in aging. Furthermore, several genes involved in acetyl-CoA metabolism are downregulated by Gis1.

## Introduction

**Figure 1:**
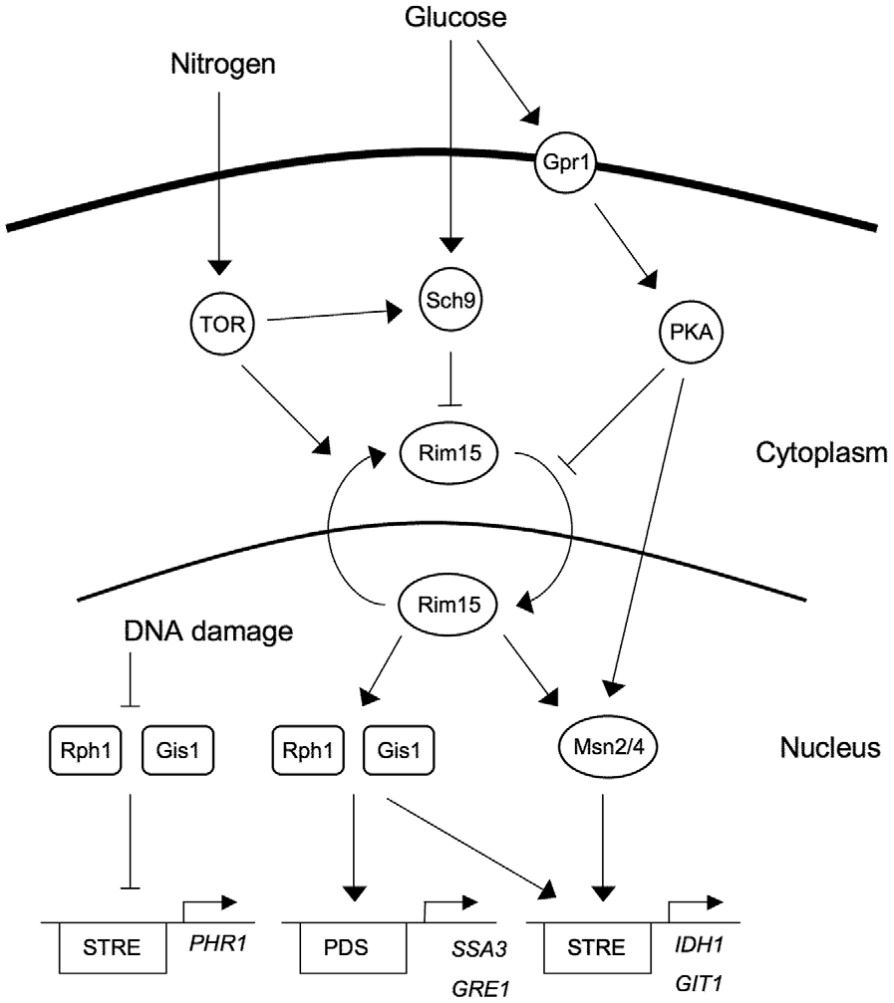
Nutrient signaling pathways in yeast. Arrows represent activation and cross-bars represent inhibition. The TOR, PKA and Sch9 pathways transmit nutrient signals to Gis1, Msn2 and Msn4 by controlling the nuclear localization of the Rim15 protein kinase, but there is evidence that the PKA and Sch9 pathways also transmit signals that are independent of Rim15. Gis1, Msn2 and Msn4 activate gene expression through PDS and STRE motifs in the promoters of their target genes. The DNA damage checkpoint pathway inhibits Gis1 and Rph1, which repress the *PHR1* gene through a STRE motif in its promoter. Our results presented here show that Rph1 also functions together with Gis1 downstream of the nutrient sensing pathways.

Nutrient limitation, also known as caloric restriction or dietary restriction, can extend both the replicative and chronological lifespan of eukaryotes as diverse as yeast, nematodes, flies, rodents, and primates [1]–[5]. These effects are mediated by the conserved nutrient sensing TOR, AKT/Sch9 and RAS/cAMP pathways, and reduced signaling by these pathways can delay aging even if nutrients are present. Yeast mutants with impaired nutrient sensing, such as *tor1*, *sch9*, or *ras2* mutants, therefore have an extended lifespan [6]–[8], and rapamycin, an inhibitor of the TOR kinase, can extend the lifespan of mice [9]. In the budding yeast *Saccharomyces cerevisiae*, the nutrient sensing pathways negatively regulate the activity and nuclear localization of the Rim15 protein kinase [10]–[13]. Rim15 in turn is thought to activate the transcription factors Gis1, Msn2 and Msn4, which turn on genes that are needed for long term survival (Figure 1). Accordingly, *rim15*, *gis1*, *msn2* and *msn4* mutations are epistatic over *tor1*, *sch9* and *ras2* mutations, and reverse the lifespan-extending phenotypes of the latter [14].

Msn2 and Msn4 are two closely related C_2_H_2_ zinc finger proteins that were cloned as dosage suppressors of the Snf1 protein kinase [15]. They bind to the STRE (STress Response Element) motif AGGGG in the promoters of stress induced genes and activate their transcription [16]–[18]. Gis1 is also a C_2_H_2_ zinc finger protein. It was isolated as a dosage suppressor of a *snf1 mig1 srb8* triple mutant [19], but was later found to regulate gene expression after glucose depletion, when yeast cells shift their metabolism from fermentation of glucose to oxidation of ethanol. This transcriptional response is called the diauxic shift, and affects the expression of more than 2,000 genes in yeast [20]. Induction of several genes at the diauxic shift is dependent on Gis1, which acts through a PDS (Post Diauxic Shift) motif, T(A/T)AGGGAT, that is present in the promoters of these genes [21]. Gis1 is also required for the induction of several mid-late and late genes during sporulation [22].

Budding yeast also has a fourth related C_2_H_2_ zinc finger protein, Rph1, whose sequence is 34% similar to that of Gis1. The zinc fingers of Gis1 and Rph1 are almost identical, which suggests that they should bind to similar DNA motifs. Rph1 was cloned as a repressor of the *PHR1* gene encoding photoreactivation lyase [23]. Rph1 and Gis1 redundantly repress *PHR1*, and also the *DPP1* gene encoding diacylglycerol pyrophosphate phosphatase [24]. It is not clear what the roles of Gis1 and Rph1 as repressors of *PHR1* and *DPP1* have in common with each other, or with the role of Gis1 as an activator in the PDS response. However, several PDS motifs are present in the *DPP1* promoter [24], and a STRE motif is found in *PHR1* [23], and there is evidence that the two proteins act through these motifs. Repression by Gis1 and Rph1 thus seems to be mediated at least in part by the same motifs as activation by Gis1. Furthermore, several studies have shown that Rph1 is able to bind the STRE motif [25]–[27].

Gis1 and Rph1 also contain JmjN and JmjC domains, which were discovered during a study of Gis1 [28]. These domains are found in many eukaryotic proteins, and possess histone demethylase activity, with the catalytic site in the JmjC domain [29]. Paradoxically, while Gis1 appears to have unique functions not shared by Rph1, only the latter has been shown to be an active histone demethylase. Rph1 demethylates di- and trimethylated lysine 36 on histone H3 (H3K36me2 and H3K36me3), and surprisingly also H3K9, which is not methylated in yeast [30]–[33]. As for Gis1, there have been indications that it may demethylate H3K36me2 and H3K36me1 [33], but there is also data suggesting that it is inactive [34]. The Gis1 JmjC domain has a missense mutation in a key residue, which supports the notion that it is inactive [35]. The JmjC domain is not required for transcriptional activation by Gis1 [22].

It is not clear which genes among the targets of Gis1, Msn2 and Msn4 that mediate the effect on aging. One proposed mechanism for life span extension is induction of oxidative stress response genes, such as *SOD2* [36]–[38]. It has also been suggested that reduced nutrient signaling promotes cell cycle arrest, which protects the cells against replicative stress [39]. Moreover, Gcn4-mediated depletion of ribosomal proteins has been implicated in life span extension [40]. Recent work has further shown that the metabolism of glycerol and in particular acetate is important for aging. Yeast cells transiently form acetic acid as glucose is depleted, and acetic acid induced mortality has been proposed to be the primary mechanism of chronological aging [41]. Conversely, glycerol protects yeast cells against stress, and the glycerol biosynthetic genes *GPD1*, *GPD2* and *RHR2* are upregulated in *sch9*, *tor1* and *ras2* mutants, resulting in higher glycerol levels [42]. Furthermore, when these genes were deleted in an *sch9* mutant, its extended lifespan phenotype was reversed [42]. Unlike acetic acid, the elevated glycerol levels persist into stationary phase.

Nutrient limitation also increases the replicative lifespan, *i.e.* the number of times that a cell can divide. Replicative aging has been linked to the accumulation of extrachromosomal rDNA circles (ERCs) in yeast [43]. ERC formation is controlled by several genes. One is *SIR2* which encodes the founding member of the sirtuin histone deacetylases, enzymes that promote longevity in all eukaryotes examined so far [5]. It is thought that the main effect of Sir2 on ERC accumulation is due to its role in silencing of rDNA, since rDNA transcription promotes ERC formation. Interestingly, the effect of caloric restriction and TOR signaling on the replicative lifespan is mediated by Msn2 and Msn4, which bind to STRE elements in the *PNC1* promoter and activate it [44]. The Pnc1 protein in turn activates Sir2 by degrading nicotinamide, an inhibitor of Sir2. ERC formation is not important for aging in multicellular eukaryotes, so Sir2 must also act in other ways to promote longevity [5]. Consistent with this, replicative aging in yeast is also associated with loss of silencing at subtelomeric loci due to increased histone acetylation [45], and elevated histone expression that enhances silencing causes life span extension [46]. These findings point to a more general role of transcriptional deregulation due to loss of silencing in replicative aging.

Several microarray studies have examined gene expression in yeast nutrient signaling mutants. One study compared gene expression in log phase and after the diauxic shift in wild type, *rim15*, *gis1*, and *msn2 msn4* double mutants [47]. Most of the 150 genes that required Rim15 for induction during the diauxic shift also required Gis1 or Msn2/4, suggesting that Gis1 and Msn2/4 mediate the effects of Rim15. Most of these genes had either STRE or PDS motifs in their promoters. A second study examined the effects of the PKA and Sch9 pathways in the absence of Msn2 and Msn4 [12]. It was found that Sch9 and PKA have both synergistic and antagonistic effects on different target genes. A third study looked at gene expression in early stationary phase in wild type, *tor1*, *ras2* and *sch9* strains [48]. Up-regulated genes were enriched for PDS and STRE motifs, suggesting that Gis1, Msn2 and Msn4 are more active in these signaling mutants. A fourth study looked at the role of Gis1 and Rim15 in glucose- and ethanol-limited cultures [49]. A previously identified set of stress-induced genes, the UES genes, was shown to depend on both Gis1 and Rim15 for induction. Additional groups of genes that were up- or down-regulated in *gis1* and *rim15* cells were identified. A fifth study identified genes that are up- or down-regulated after 2.5 days in *ras2*, *sch9* and *tor1* mutants [42]. A significant overlap was found between the three mutants both for up- and down-regulated genes.

There is no previous evidence that Rph1 functions in nutrient signaling, but Gis1 and Rph1 are both induced after glucose depletion, with Rph1 expression peaking after 3 days and Gis1 after 5 days [50]. Moreover, Rph1 is phosphorylated upon treatment with rapamycin, an inhibitor of the TOR kinase [51]. This suggested to us that Rph1 also could play a role in growth phase-dependent gene expression. To test this, we used microarrays to study gene expression in a wild type strain, *gis1* and *rph1* single deletions, and a *gis1 rph1* double deletion. Our results show that Rph1 is involved in growth phase-dependent control of gene expression and reveal that Gis1 and Rph1 function both as repressors and activators, on overlapping sets of genes as well as on distinct targets. Furthermore, we see significant effects on both acetate and glycerol accumulation in the mutants. Consistent with this, we find that several genes involved in glycerol and acetate metabolism are regulated by Gis1 and Rph1. In addition, several genes involved in acetyl-CoA metabolism are downregulated by Gis1. Taken together, our findings provide possible links between nutrient signaling, metabolic regulation and the control of aging in yeast.

## Results

### Gis1 and Rph1 jointly regulate acetate and glycerol accumulation after the diauxic shift

**Figure 2:**
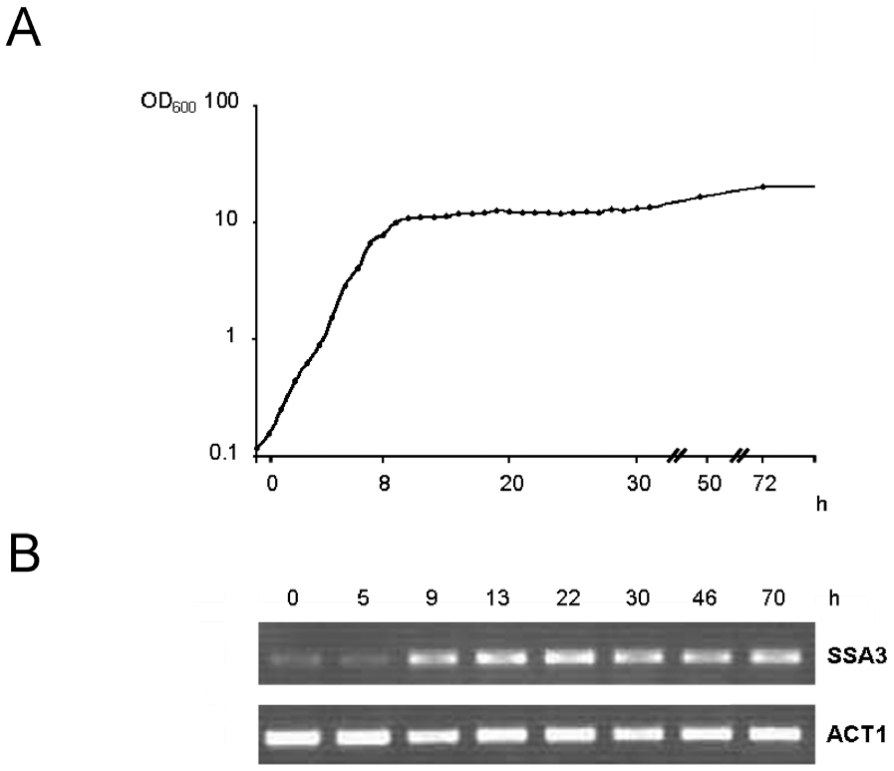
Growth curves and *SSA3* gene expression. (A) Growth curve for yeast strain BY4742 grown in batch culture for three days. (B) *SSA3* expression over time. Samples were harvested at an OD_600_ of 0.4 (time zero) and at different later time points as indicated in the figure. The amount of *SSA3* mRNA at each time point was measured using reverse transcriptase-PCR with specific oligonucleotide primers. The induction of *SSA3* expression coincides with the diauxic shift-associated decrease in the growth rate. Expression of the *ACT1* gene encoding actin was included as a control.

We studied gene expression under twelve distinct conditions: four different yeast strains (wild type, *gis1* and *rph1* single deletion mutants, and a *gis1 rph1* double deletion mutant) at three different time points reflecting different growth phases (log phase, diauxic shift and early stationary phase, see Figure 2A). The log phase cells were taken at an OD_600_ of 0.4 from a culture kept in continuous exponential growth for 24 h by repeated dilutions. To determine the onset of the diauxic shift accurately, we examined expression of the *SSA3* gene [21] by reverse transcriptase-PCR. Our results show that the gene was fully induced 9 h after the log phase time point (Figure 2B). Accordingly, samples for the microarray experiment was taken at this point. The final time point chosen was after three days of growth, at the transition between late PDS phase and early stationary phase, when the ethanol that was formed after the diauxic shift had been fully consumed (see below). All experiments were performed using biological triplicates. To validate the microarray results, the expression of selected genes from different clusters (see below) were also measured by quantitative RT-PCR (qPCR). As seen in Table S1, the qPCR data was in good agreement with the array data, though generally of lower significance, which in a few cases caused the qPCR p-value to fall above the 0.02 threshold when the array p-value was below this threshold.

**Figure 3:**
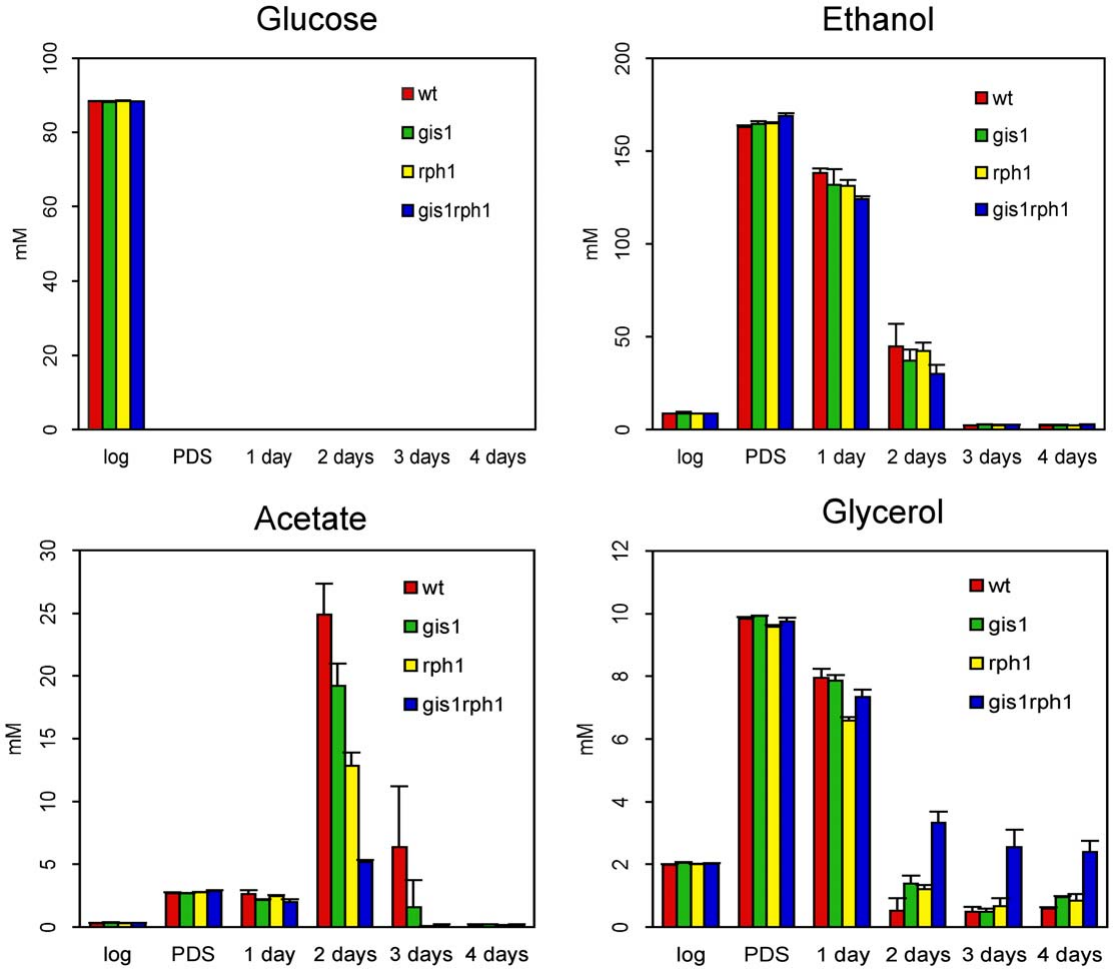
Effects of Gis1 and Rph1 on extracellular metabolite concentrations. The concentrations of glucose, ethanol, acetate and glycerol at different time points in culture supernatants from the four strains (wild type, *gis1*, *rph1* and *gis1 rph1*). The concentrations were determined using HPLC as described in Materials and Methods. The error bars show the standard deviations of three independent replicates.

We first checked if the mutants had any obvious phenotypes affecting growth or metabolism. No significant differences were seen between the growth rates of the four strains. To look for effects on the metabolism, we analyzed culture supernatants taken at different time points for glucose, ethanol, acetate and glycerol (see Materials and Methods). As expected, glucose was rapidly consumed by all four strains, being fully depleted after the diauxic shift (Figure 3). Ethanol accumulation and consumption was also similar in all four strains, with all ethanol having been consumed after three days of culture. However, a significant effect was seen on acetate accumulation, which was reduced in the mutants, particularly in the *gis1 rph1* double mutant (Figure 3). Furthermore, the latter strain instead had a significant amount of glycerol in the supernatants from days 2–4, which was almost absent in supernatants from the wild type strain. We conclude that Gis1 and Rph1 together function to enhance acetate accumulation and reduce glycerol accumulation after the diauxic shift.

### Gis1 and Rph1 are both involved in growth phase-dependent gene regulation

**Figure 4:**
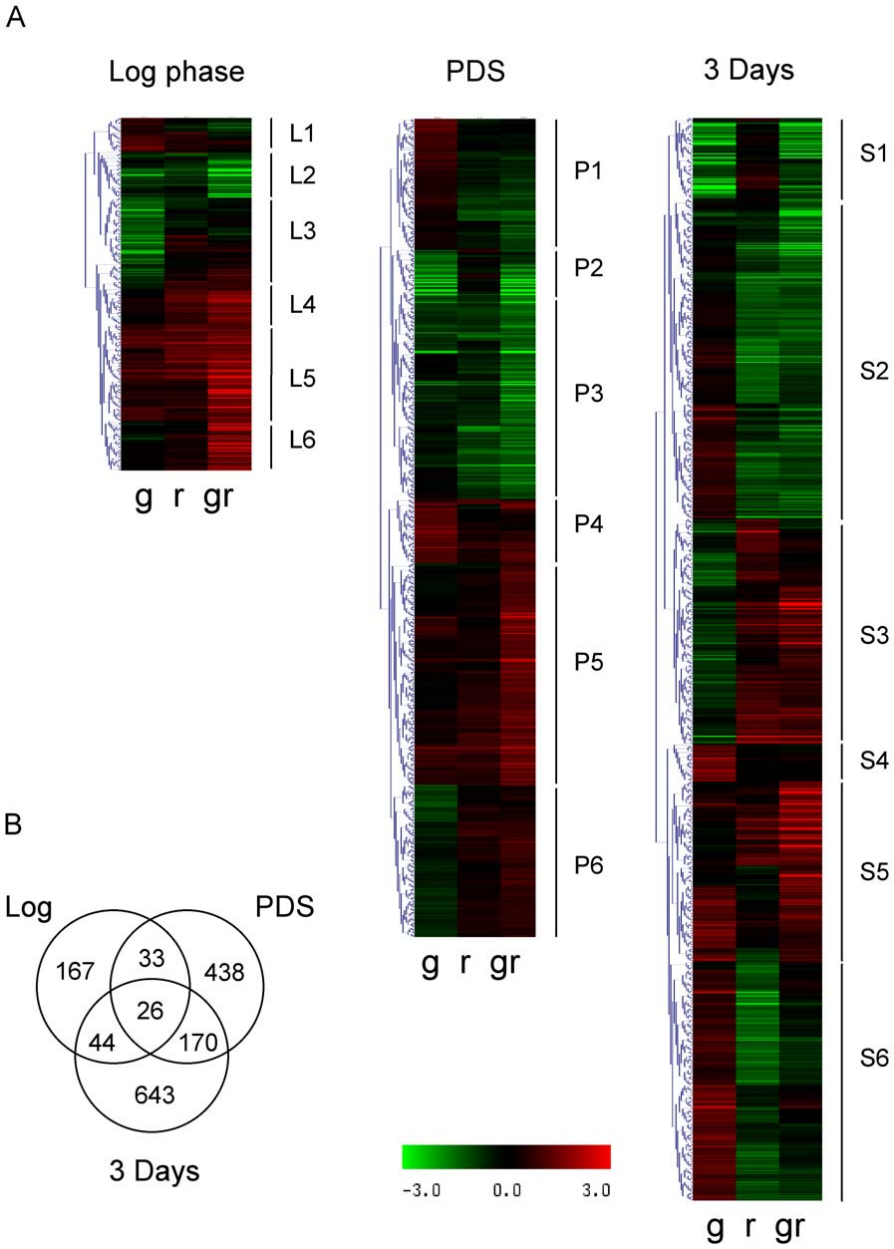
Effects of Gis1 and Rph1 on gene expression. (A) Clustering of differential gene expression profiles at each growth stage. The heat maps show the log ratios in the following contrasts: *gis1 vs.* wild type (g), *rph1 vs.* wild type (r) and *gis1 rph1 vs.* wild type (gr). Only genes that had a significant differential expression in any of the pairwise strain comparisons were included. Selected clusters are highlighted, with details given in Tables 1–3. (B) The number of differentially expressed genes in comparisons between different strains for each growth stage. Only 26 genes are differentially expressed at all three time points, and most genes are only differentially expressed at one time point.

Since we were interested in the roles of Gis1 and Rph1, we looked for genes whose expression is affected by a deletion of *GIS1* and/or *RPH1*, *i.e.* whose expression differ significantly in a pairwise comparison of any two strains at any of the three time points (Figure 4A). Only genes that were up- or downregulated at least 2-fold, with p<0.01 were considered. In total, 1,521 genes that fulfilled these criteria were found. Significantly, we found that different sets of genes are differentially expressed in the log phase, after the diauxic shift and after three days (Figure 4B), indicating that Gis1 and Rph1 play different roles at different stages of growth.

To identify co-regulated genes, we carried out hierarchical clustering on the expression profiles of all 1,521 Gis1- and/or Rph1-regulated genes at each time point (Figure 4A). In total, 18 clusters were identified, six at each time point (L, P and S stand for Log phase, PDS phase and Stationary phase). Eleven clusters had clear and easily explained expression patterns. For example, if a gene has negative log ratios in the contrasts *gis1 vs.* wild type and *gis1 rph1 vs. rph1*, this suggests that the gene is upregulated by Gis1. Three clusters (L3, P4 and S4) showed significant effects only in the *gis1* mutant, but not in the *rph1* mutant or the double mutant. A possible interpretation is that *rph1* is epistatic over *gis1* for its effect on these genes. The remaining four clusters (L1, P1, P6, and S3) had expression patterns that were harder to interpret. Genes in these clusters typically showed small effects in response to single and double deletions, and frequently in opposite directions.

The 18 clusters are listed in Tables 1–3. For each cluster, we looked for enriched functions using both Gene Ontology and manual annotations, and for enrichment of STRE and PDS motifs in the promoters. We also computed two parameters that characterize the mode of regulation (see Materials and Methods). The *Gis1/Rph1 ratio* shows whether Gis1 or Rph1 has the largest effect. A positive number indicates a larger effect of Gis1 and a negative number a larger effect of Rph1. The *redundancy coefficient* indicates to what extent Gis1 and Rph1 cooperate in regulation. A value of 1 means complete redundancy, *i.e.* a deletion of both is required for any effect, whereas a value of 0 suggests a synergistic regulation, where a partial effect is seen in the single deletions. Some clusters have a negative redundancy coefficient, indicating a larger effect in one or both single deletions than in the double deletion. The modes of regulation found were: 1) activation by Gis1 alone, 2) repression by Gis1 alone, 3) activation by Rph1 alone, 4) repression by Rph1 alone, 5) redundant repression, 6) synergistic activation, and 7) synergistic repression. There were no clusters that were redundantly activated, but individual genes with this pattern were found.

**Table 1:** Expression details for the 6 log phase (L) gene clusters identified in Figure 4A.

Cluster	Interpretation	Genes	Enriched functions	Examples of genes	Redundancy	Gis1/Rph1 ratio	STRE^a^ (nr/pval)	PDS^b^ (nr/pval)
L1	Unclear	25		*ACE2, ADH5, CDC5, GDH1, IRA1*	1.28	0.43	0.44/8.1e-1	0.2/6.3e-1
L2	Activated by Gis1 and Rph1	29	**Phosphate metabolism.**	*MET6, MET14, * ***PHO5, PHO8, PHO11, PHO81, PHO84, PHO89***	0.23	0.15	0.21/1.8e-3^c^	**0.38/4.5e-2**
L3	Down only in the *gis1* mutant^d^	51	**Sulfur metabolism.** Ribosome biogenesis.	***MET1, MET2, MET13, MET28,*** * NOP7, NOP16, RRP12, STR3*	−0.32	0.84	0.57/4.5e-1	0.12/4.4e-1
L4	Repressed by Rph1 (mainly)	32	Glutathione metabolism.	*GND2, GTT1, GPX1, HYR1*	0.27	−0.53	**1.16/1.5e-3**	**0.38/3.6e-2**
L5	Synergistically repressed	72	Glycolysis. Glycerol and glycogen metabolism. Pentose-P pathway. Stress response.	*ALD4, CTT1, GCY1, GDB1, GPH1, GSY2, HAP4, HOR2, HSP12, HSP12, HXK1, HXT6, MSN4, TPK2, TRR2, ZWF1*	−0.13	−0.13	**1.81/6.1e-10**	0.29/6.4e-2
L6	Redundantly repressed	37		*GTT2, HSP30, MRK1, XBP1*	0.80	−0.20	**1.32/3.6e-4**	0.35/1.3e-1

Strongly enriched functions and genes are shown in bold face.

a In the group of all promoters, there are on average 0.47 STRE motifs/promoter. Enrichments significant at p<0.05 are shown in bold face.

b In the group of all promoters, there are on average 0.15 PDS motifs/promoter. Enrichments significant at p<0.05 are shown in bold face.

c Significantly lower occurrence of the motif than in the reference group of all promoters.

**Table 2 pone-0031577-t002:** Expression details for the 6 PDS phase (P) gene clusters identified in Figure 4A.

Cluster	Interpretation	Genes	Enriched functions	Examples of genes	Redundancy	Gis1/Rph1 ratio	STRE^a^ (nr/pval)	PDS^b^ (nr/pval)
P1	Unclear	99	Nutrient and stress signaling.	*BCY1, CDC16, CDC55, HAC1 MTH1, MIG1, PCL5, PHO2, RIM101, SCH9, SKN7, XBP1*	1.16	0.19	**1.04/1.0e-5**	0.16/9.6e-1
P2	Gis1-activated	31	Stress response.	*GDH3, GND2, GRE1, SSA3, TKL2*	0.05	1.11	**1.32/5.4e-4**	**0.48/2.6e-2**
P3	Activated by Gis1 and Rph1	154	Amino acid metabolism. Intermediate metabolism. Nutrient and stress signaling.	*ALD2, ALD3, ARG80, BMH2, GIS3, GPA2, GRE2, GUT1, MET2, MET6, PHO8, PH084, PHO89, PLC1, SRY1, STE11, ZWF1*	0.19	−0.26	**0.83/2.0e-5**	**0.31/6.7e-4**
P4	Up only in *gis1* ^d^	49		*BAT1, GUT2, PHO80, RNR1*	−0.97	0.76	**0.82/3.3e-2**	0.12/4.4e-1
P5	Repressed by Gis1 and Rph1	163	**Ribosome biogenesis.** Cell cycle control, DNA and RNA polymerases.	**22 ** ***RPL*** ** and 17 ** ***RPS*** ** genes** *CDC12, CDC21, CDC45, CLN1, CLN2, POL1, POL2, POL30, RPB11, RPC10, RPC11*	0.38	−0.13	0.53/3.8e-1	0.13/3.9e-1
P6	Unclear	112	**Ribosome biogenesis.**	**27 ** ***RPL*** ** and 20 ** ***RPS*** ** genes**	1.25	0.16	0.42/4.2e-1	0.14/6.7e-1

Strongly enriched functions and genes are shown in bold face.

a In the group of all promoters, there are on average 0.47 STRE motifs/promoter. Enrichments significant at p<0.05 are shown in bold face.

b In the group of all promoters, there are on average 0.15 PDS motifs/promoter. Enrichments significant at p<0.05 are shown in bold face.

**Table 3:** Expression details for the 6 early stationary phase (S) gene clusters identified in Figure 4A.

Cluster	Interpretation	Genes	Enriched functions	Examples of genes	Redundancy	Gis1/Rph1 ratio	STRE^a^ (nr/pval)	PDS^b^ (nr/pval)
S1	Gis1-activated	57	Stress response.	*ALD2, ALD3, GND2, GRE1, SSA3*	0.00	1.06	**0.98/3.5e-4**	**0.60/8.0e-5**
S2	Activated by Rph1 (mainly)	246	Cell cycle control and DNA polymerases. Stress response.	*CDC14, CDC21, CDC28, CDC45, CTT1, GRE2, GRE3, GDH3, HSP31, PDC5, PDC6, POL1, POL30, SSA1, SSA2, SWI5*	0.45	−0.52	0.59/6.0e-2	0.22/8.7e-2
S3	Unclear	158	Ribosome biogenesis.	8 *RPL* and 6 *RPS* genes. CDC55, *PHO84, PHO89, POL12, TPK1*	0.87	−0.14	0.48/8.9e-1	0.22/8.7e-2
S4	Up only in *gis1* c	25		*GAT1, GDH1, HXT4, SGA1*	−4.68	1.22	**1.32/7.9e-3**	0.20/6.9e-1
S5	Repressed by Gis1 and Rph1	123	Ribosome biogenesis. Cell wall biosynthesis and turnover.	5 *RPL* and 5 *RPS* genes. *CLN2, CLN3, CTS1, DSE2, DSE4, EGT2, ERG3, ERG11, ERG25, EXG1, FBP1, HOR2, PCL9*	0.39	−0.02	**0.63/3.7e-2**	0.20/2.8e-1
S6	Gis1-repressed Rph1-activated	190	Carboxylic acid metab process. Amino acid biosynthesis. Lipid biosynthesis. Methyl group metabolism.	*AAT1, ACH1, ACO1, ACS2, ERG1, ERG6, ERG13, ERG26, ERG27, GUS1, HMG1, IDH1, IDH2, ILV1, LCS1, MAE1, PRO1, PRO3, SAH1, SDH3, SPE2*	0.50	−0.13	0.56/1.7e-1	0.13/3.0e-1

Strongly enriched functions and genes are shown in bold face.

a In the group of all promoters, there are on average 0.47 STRE motifs/promoter. Enrichments significant at p<0.05 are shown in bold face.

b In the group of all promoters, there are on average 0.15 PDS motifs/promoter. Enrichments significant at p<0.05 are shown in bold face.

c No effect was seen in the double mutant.

It should further be noted that the same gene may occur in clusters from different time points, and that there are significant correlations between clusters at different time points due to this (Table 4). It is not, however, the case that the same groups of genes form similar clusters at all three time points. Instead, we see a partial overlap between clusters. For example, clusters S2 and S5 overlap with both L5 and P5. Of these, L5, P5 and S5 are all repressed by both Gis1 and Rph1, and L5 and S5 are enriched for STRE motifs (Tables 1–3). Similarly, clusters P2, P3 and S1 show a significant overlap, and they are all activated by Gis1 and/or Rph1. All three clusters are also enriched for both STRE and PDS motifs (Tables 2 and 3).

**Table 4:** Overlap between clusters in Tables 1–3 from different time points.

Cluster	P1 (106)	P2 (35)	P3 (169)	P4 (52)	P5 (177)	P6 (124)
L1 (26)	0	0	0	1 0.19	1 0.52	1 0.4
L2 (34)	0	0	**10 9.1e-9**	1 0.24	1 0.61	0
L3 (51)	1 0.57	0	2 0.39	1 0.34	0	0
L4 (33)	1 0.42	**3 7.2e-4**	**5 1.5e-3**	0	1 0.6	0
L5 (74)	**5 7.2e-3**	1 0.33	4 0.13	**5 3.0e-04**	2 0.61	0
L6 (38)	**4 3.3e-3**	1 0.19	**5 2.9e-3**	**3 3.4e-3**	2 0.28	0

Cluster	S1 (62)	S2 (261)	S3 (184)	S4 (30)	S5 (147)	S1 (62)
L1 (26)	0	0	0	1 0.11	2 0.12	2 0.19
L2 (34)	0	1 0.76	2 0.25	0	2 0.18	1 0.65
L3 (51)	0	6 0.016	1 0.77	0	3 0.11	3 0.2
L4 (33)	**4 2.6e-4**	1 0.75	3 0.067	1 0.14	1 0.53	0
L5 (74)	2 0.16	**9 2.8e-3**	3 0.36	2 0.046	**6 6.6e-3**	3 0.39
L6 (38)	**3 5.7e-3**	1 0.79	2 0.3	2 0.013	3 0.055	0

Cluster	S1 (62)	S2 (261)	S3 (184)	S4 (30)	S5 (147)	S1 (62)
P1 (106)	0	2 0.93	**10 8.5e-4**	0	5 0.095	2 0.84
P2 (35)	**17 2e-26**	**8 6e-5**	0	0	1 0.56	0
P3 (169)	**17 1.6e-13**	8 0.38	**23 3.0e-10**	1 0.55	3 0.75	2 0.97
P4 (52)	1 0.4	4 0.16	1 0.78	1 0.22	6 0.0011	5 0.02
P5 (177)	1 0.82	**20 2.8e-5**	7 0.24	1 0.57	**23 7.3e-12**	10 0.042
P6 (124)	0	7 0.24	5 0.28	0	2 0.78	0.52

In each comparison the number of overlapping genes and the hypergeometrical p-value is shown. Entries that are significant at p<0.01 are shown in bold. The number of genes in each cluster is shown in parenthesis.

### Functionally distinct groups of genes are differentially regulated by Gis1 and Rph1

In log phase cells, cluster L2, which is synergistically activated by Gis1 and Rph1, is highly enriched for genes involved in phosphate metabolism, and cluster L3 which is downregulated in the *gis1* mutant is highly enriched for genes involved in sulfur metabolism. The L2 genes are slightly enriched for PDS motifs but contain fewer STRE motifs than expected (Table 1). The L3 genes are not enriched for either motif. Cluster L4, which is repressed mainly by Rph1, encodes several enzymes involved in glutathione metabolism and the defense against oxidative stress. Cluster L5 encodes enzymes involved in glycerol and glycogen metabolism and the pentose phosphate pathway, but also some stress response genes. Interestingly, the transcription factors Msn4 and Hap4, which activate stress response genes and respiratory genes, respectively, are encoded by genes in cluster L5. Cluster L6, which is redundantly repressed, is not obviously enriched for any group of genes, but the transcription factor Xbp1 (see below) is encoded by this cluster. L4, L5 and L6 are all strongly enriched for STRE motifs and L4 is also enriched for PDS motifs. In conclusion, genes involved in phosphate and sulfur metabolism are upregulated by Gis1 and Rph1 in the log phase, but this activation is largely indirect as evidenced by the lack of STRE motifs and only a slight enrichment of PDS motifs. Genes involved in oxidative carbon metabolism and the defense against oxidative stress are downregulated, and they are likely direct targets of Gis1 and Rph1 as evidenced by an excess of STRE motifs in their promoters.

The PDS phase cluster P1 (Table 2) encodes several proteins involved in nutrient and stress signaling, such as Bcy1, Mth1, Mig1, Pcl5, Sch9 and Skn7. These genes are enriched for STRE motifs, so they are likely direct targets of Gis1 and Rph1, but their modes of regulation are not easily interpreted. Cluster P2, which is activated by Gis1 alone and enriched for both STRE and PDS motifs, contains well-studied PDS response genes such as *SSA3* and *GRE1* [18]. Cluster P3, which is activated by Gis1 and Rph1, contains a number of genes involved in amino acid metabolism and nutrient and stress signaling. Both STRE and PDS motifs are highly enriched in cluster P3. Cluster P5, which is repressed by Gis1 and Rph1, is highly enriched for ribosomal protein genes, but also for genes encoding cell cycle regulators and RNA and DNA polymerases. P5 is thus made up of genes whose expression is strongly associated with cell growth. Cluster P6, whose expression is harder to interpret, contains even more ribosomal protein genes. Neither P5 nor P6 is enriched for STRE or PDS motifs, but P5 is enriched for the binding motifs of Mbp1 and Swi4, two transcription factors that control gene expression during the cell cycle [52]. In conclusion, genes involved in nutrient and stress signaling, stress response genes, and genes involved in amino acid metabolism are upregulated in PDS-phase cells, and they are likely direct targets of Gis1 and Rph1. Genes associated with cell growth, in particular ribosomal protein genes, are downregulated, but the lack of STRE and PDS motifs suggests that they are not direct targets of Gis1 or Rph1.

After 3 days, the Gis1-activated cluster S1 strongly overlaps with cluster P2, which contained the Gis1-activated PDS response genes (Table 4). Like P2, cluster S1 is strongly enriched for both STRE and PDS motifs (Table 3). Cluster S2, with 246 genes, is the largest cluster in our analysis, and contains genes that are activated mainly by Rph1. They include stress response genes, but surprisingly also genes encoding cell cycle regulators and DNA polymerase subunits. One of the latter is the PCNA gene *POL30*, which is interesting since Rph1 also is involved in the DNA damage response, as a repressor of the DNA repair gene *PHR1* [23]. In any case, cluster S2 is not enriched for either STRE or PDS motifs, unlike *PHR1*, whose promoter contains an Rph1-binding STRE motif [23]. The small cluster S4 contains genes that are upregulated only in the *gis1* mutant but not the double mutant, and is enriched for STRE motifs. Cluster S5, which is repressed by Gis1 and Rph1, contains a few ribosomal subunit genes, but far fewer than clusters P5 and P6 which were repressed by Gis1 and Rph1 in the PDS phase. Instead, cluster S5 is enriched for genes involved in cell wall biosynthesis and turnover, and it also differs from P5 and P6 in that it is enriched for STRE motifs. We note that the absence of an effect of our mutants on the ribosomal protein genes after 3 days does not necessarily mean that they are no longer repressed by Gis1 and Rph1. It could be that some other mechanism contributes to the silencing of these genes in stationary phase, thus making a repression by Gis1 and Rph1 redundant.

The most interesting cluster is cluster S6 (Table 3), which is subject to an unusual mode of regulation, being repressed by Gis1 but activated by Rph1. This cluster encodes a number of enzymes involved in acetyl-CoA synthesis and the further metabolism of acetyl-CoA in the TCA cycle, amino acid biosynthesis and lipid biosynthesis. It is also enriched for genes involved in the methyl cycle and methyl group metabolism. The promoters of the genes in cluster S6 are not enriched for either STRE or PDS motifs, so it is likely that their regulation by Gis1 and Rph1 is indirect. We note that these genes are enriched for binding sites of Xbp1, a repressor that is induced by stress and starvation [53]. *XBP1* is one of the genes that are repressed by Gis1 and Rph1 (cluster L6), and the *XBP1* promoter contains five STRE motifs. It is therefore possible that the regulation of cluster S6 genes by Gis1 and Rph1 to some extent is mediated by Xbp1. However, many genes in cluster S6 lack Xbp1 sites, so this cannot be the only explanation.

### Gis1 and Rph1 jointly regulate targets of the PKA, Sch9 and TOR nutrient signaling pathways

Since Gis1 functions downstream of the RAS/cAMP, Sch9 and TOR pathways [12], [21], [42], [39], [47], we paid special attention to genes that are regulated by these three pathways. To this end, we studied how several previously identified groups of genes respond to a deletion of Gis1 and/or Rph1. The groups of genes examined included the Rim15-activated [47], genes that are up- or downregulated in the presence of rapamycin [54], genes that are up- or downregulated in *ras2*, *tor1* and *sch9* mutants [42] and genes that are up- or downregulated in a *gis1* mutant [49]. We also included the so-called UES genes [49] and the class II genes of Roosen et al. [12], which are repressed by the RAS/cAMP pathway but activated by overexpression of Sch9.

The average response of each group of genes (direction and p-value) in our mutants is shown in Table 5. In log phase cells, we found that the class II genes, the Rim15-activated genes, the rapamycin-activated genes, the Ras2-repressed genes, and the Gis1-repressed genes are all significantly upregulated in the *gis1* mutant, the *rph1* mutant, and the double mutant. The UES genes, the Sch9-repressed genes, the Tor1-repressed genes, and, interestingly, the Gis1-activated genes are significantly upregulated in the *rph1* and double mutants, but not in the *gis1* mutant. Conversely, the Ras2-activated genes are downregulated in all three mutants, and the Tor1-activated genes are downregulated in the *gis1* mutant (Table 5). We conclude that there is a strong correlation between genes that are repressed by one or several nutrient sensing pathways and genes that are repressed by Gis1 and Rph1 in the log phase. This repression makes sense since nutrients are abundant in log phase cultures, but the fact that repression of this group of genes is mediated by both Gis1 and Rph1 is a new finding.

**Table 5:** Effects of Gis1 and Rph1 on the expression of genes regulated by nutrient signaling.

Genes [ref] (number)		Log phase			PDS phase			3 days	
Genetic contrast	gis1-wt	rph1-wt	gis1rph1-wt	gis1-wt	rph1-wt	gis1rph1-wt	gis1-wt	rph1-wt	gis1rph1-wt
Class II genes [12] (289)	**↑ <1e-30**	**↑ <1e-30**	**↑ <1e-30**	↓ 2.3e-1	**↓ 3.5e-4**	**↓ 4.2e-9**	↓ 1.5e-1	↓ 4.8e-1	**↓ 1.8e-3**
Rim15 activated [47] (57)	**↑ 4.7e-3**	**↑ 2.3e-8**	**↑ 6.3e-7**	↓ 9.8e-1	**↓ 2.1e-4**	**↓ 5.1e-3**	↓ 4.3e-2	↓ 7.8e-1	↓ 3.5e-2
Rap repressed [54] (109)	↓ 2.8e-1	↓ 2.2e-2	↑ 8.2e-1	↓ 3.0e-1	↑ 1.8e-1	**↑ 4.1e-7**	↓ 4.6e-1	↑ 2.5e-1	**↑ 4.9e-8**
Rap activated [54] (152)	**↑ 1.8e-6**	**↑ 2.2e-11**	**↑ 1.5e-5**	↑ 7.1e-1	**↓ 2.0e-3**	**↓ 9.5e-4**	↑ 4.3e-2	↓ 5.1e-2	↓ 1.3e-1
Sch9 repressed [42] (142)	↑ 2.1e-2	**↑ 9.5e-6**	**↑ 4.5e-7**	↓ 1.1e-2	↓ 6.8e-1	↓ 4.0e-2	↑ 7.6e-1	↓ 8.2e-2	↓ 2.7e-2
Ras2 repressed [42] (321)	**↑ 8.5e-3**	**↑ 1.2e-7**	**↑ 9.8e-5**	↓ 5.8e-1	↑ 6.8e-2	↑ 9.7e-1	**↑ 1.0e-4**	**↓ 3.4e-5**	**↓ 1.3e-4**
Tor1 repressed [42] (129)	↑ 5.2e-1	**↑ 6.3e-4**	**↑ 1.2e-4**	↓ 3.2e-2	↓ 2.6e-1	↓ 8.1e-1	↓ 2.5e-1	↓ 1.1e-1	↓ 1.9e-1
Sch9 activated [42] (117)	↑ 2.7e-1	↑ 8.9e-1	↑ 1.1e-1	↑ 1.8e-1	**↑ 2.8e-5**	**↑ 5.0e-8**	↑ 3.6e-1	↓ 2.5e-1	**↓ 2.0e-3**
Ras2 activated [42] (349)	**↓ 1.3e-5**	**↓ 2.0e-3**	**↓ 7.6e-3**	↑ 1.8e-1	**↑ 5.9e-3**	↑ 1.5e-2	**↓ 1.5e-5**	**↑ 3.4e-4**	↓ 4.8e-2
Tor1 activated [42] (59)	**↓ 3.5e-3**	↑ 1.7e-1	↑ 8.3e-1	↑ 1.7e-1	**↑ 4.7e-4**	**↑ 7.9e-4**	**↑ 6.5e-3**	**↓ 4.6e-3**	↓ 2.5e-2
UES genes [49] (17)	↑ 2.0e-1	**↑ 6.1e-4**	**↑ 9.9e-4**	**↓ 5.9e-3**	**↓ 1.5e-3**	**↓ 8.8e-4**	**↓ 5.0e-9**	**↓ 7.7e-4**	↓ 6.8e-1
Gis1 activated [49] (23)	↑ 6.6e-1	**↑ 4.6e-5**	**↑ 3.2e-5**	**↓ 3.1e-4**	↓ 9.5e-1	**↓ 9.0e-3**	↓ 3.1e-1	↓ 2.2e-1	**↓ 3.2e-3**
Gis1 repressed [49] (25)	**↑ 1.2e-10**	**↑ 1.3e-8**	**↑ 2.3e-13**	**↑ 6.8e-3**	↓ 1.3e-2	↓ 9.6e-1	↑ 8.7e-2	↑ 8.4e-1	↑ 9.5e-1
GO Carboxylic acid (395)	↑ 2.7e-2	**↑ 6.5e-11**	↑ 3.2e-1	↓ 8.1e-1	**↓ 3.7e-3**	↓ 2.3e-1	**↑ 3.0e-14**	**↓ 4.2e-26**	**↓ 1.1e-3**
KEGG Acetyl-CoA (32)	↑ 8.9e-2	↑ 2.1e-2	↑ 2.8e-2	↑ 8.6e-2	↓ 1.5e-1	↑ 4.2e-1	**↑ 3.1e-3**	**↓ 3.7e-6**	**↓ 6.3e-3**

The effect of each contrast on the expression of previously described groups of genes involved in nutrient signaling are shown. The overall direction of change (up or down) and Wilcoxon rank sum p-values are listed, with significant effects at p<0.01 shown in bold face. The number of genes in each group is shown in parenthesis and the reference in square brackets. The bottom rows shows the same data for 2 gene onthology groups, with the number of genes in each group in parenthesis.

Consistent with a role for Gis1 and Rph1 in nutrient signaling, we found that several groups of nutrient-repressed genes instead are downregulated in our mutants after the diauxic shift, when nutrients have been depleted (Table 5). However, this effect is less pronounced than the Gis1/Rph1-dependent log phase repression. In particular, we note that a significant effect on the class II genes, the Rim15-activated genes, and the rapamycin-activated genes is seen only in the *rph1* single and *gis1 rph1* double mutants, but not in the *gis1* single mutant. This suggests that Rph1 is more important than Gis1 for PDS induction of these genes. As expected, genes that are induced by nutrient signaling, *i.e.* the Sch9-activated genes, the Ras2-activated genes, the Tor1-activated genes, and the rapamycin-repressed genes, are instead upregulated in our mutants after the diauxic shift (Table 5). However, also in this case significant effects are seen only in the *rph1* mutant or the *rph1 gis1* double mutant. The only groups of genes whose PDS phase expression is significantly affected in the *gis1* mutant are the previously described Gis1-activated, Gis1-repressed, and UES genes [49]. After 3 days the effects are more complex, and some groups of genes are now regulated in opposite directions by Gis1 and Rph1 (Table 5). Thus, the Ras2-repressed and Tor1-activated genes are now upregulated in the *gis1* mutant and downregulated in the *rph1* mutant, whereas the Ras2-activated genes are downregulated in the *gis1* mutant and upregulated in the *rph1* mutant.

To further analyze these effects, we examined how the clusters in Tables 1–3 correlate with genes involved in nutrient signaling. As seen in Table 6, there are significant correlations between at least one of our clusters and all groups of genes with one exception only: the Ras2-activated genes [42]. In particular, clusters L4, P2 and S1 share a very similar pattern, being strongly correlated with the class II genes [12], the Sch9-repressed, Ras2-repressed, and Tor1-repressed genes [42], and the UES genes and Gis1-activated genes [49]. One would therefore expect these clusters to correlate with each other, and this is indeed the case. Clusters P2 and S1 are the two most highly correlated clusters: 17 of the 35 genes in P2 are found also in S1 (Table 4). Cluster L4 is also significantly correlated with P2 and S1 though the number of shared genes is smaller. We note that clusters P2 and S1 comprise genes that are activated mostly by Gis1, and they include the most well-studied gene in the PDS response, *SSA3*. Finally, cluster S6, which is repressed by Gis1 but activated by Rph1, shows a significant overlap with the Ras2-repressed and Tor1-activated genes, both of which also are upregulated in the *gis1* mutant and downregulated in the *rph1* mutant (Table 6).

**Table 6:** Overlap between the clusters in Tables 1–3 and genes regulated by nutrient signaling.

Cluster (genes)	L1 (26)	L2 (34)	L3 (51)	L4 (33)	L5 (74)	L6 (38)	P1 (106)	P2 (35)	P3 (169)	P4 (52)	P5 (177)	P6 (124)	S1 (62)	S2 (261)	S3 (184)	S4 (30)	S5 (147)	S6 (195)
Class II genes [12] (289)	0	1 0.79	4 0.19	**12 8.2e-9**	**15 7.1e-7**	**6 6.4e-3**	9 0.048	**14 9.9e-11**	**17 1.5e-3**	2 0.69	4 0.96	0	**21 8.6e-14**	16 0.13	7 0.73	1 0.75	12 0.032	9 0.52
Rim15 activated [47] (57)	0	0	0	2 0.034	**6 4.3e-5**	**3 4.5e-3**	1 0.61	1 0.27	5 0.016	0	0	0	1 0.43	0	2 0.49	0	0 1	4 0.094
Rap repressed [54] (109)	1 0.36	1 0.44	3 0.055	0	2 0.36	0	2 0.54	0	0	3 0.058	**9 3.0e-3**	**11 6.8e-6**	0	3 0.83	7 0.036	0	4 0.24	3 0.65
Rap activated [54] (152)	1 0.46	0	0	2 0.18	**9 5.4e-5**	1 0.6	4 0.24	1 0.57	**11 2.1e-3**	2 0.35	3 0.79	1 0.95	3 0.18	9 0.16	6 0.27	3 0.033	8 0.023	7 0.18
Sch9 repressed [42] (142)	2 0.11	1 0.53	4 0.026	**4 5.7e-3**	3 0.22	1 0.57	2 0.68	**11 1.1e-10**	**11 1.2e-3**	4 0.027	7 0.096	4 0.29	**18 4.2e-16**	10 0.062	9 0.02	1 0.49	**11 3.7e-4**	6 0.26
Ras2 repressed [42] (321)	**6 1.5e-3**	**8 2.0e-4**	4 0.25	5 0.023	5 0.31	6 0.011	3 0.91	**14 4.0e-10**	**17 4.4e-3**	**11 3.9e-5**	14 0.058	7 0.42	**22 6.3e-14**	**32 1.7e-6**	13 0.13	5 0.015	**19 1.2e-4**	**21 6.7e-4**
Tor1 repressed [42] (129)	3 0.015	**4 4.5e-3**	2 0.27	**4 4.0e-3**	2 0.44	2 0.18	4 0.16	**10 8.9e-10**	**9 6.7e-3**	3 0.086	**16 3.9e-7**	4 0.24	**16 3.3e-14**	10 0.036	**12 3.0e-4**	2 0.12	**17 4.0e-9**	6 0.2
Sch9 activated [42] (117)	0	0	1 0.61	0	2 0.39	1 0.5	0	0	0	1 0.62	**11 3.5e-4**	3 0.39	1 0.68	**13 8.5e-4**	2 0.85	1 0.42	4 0.28	4 0.48
Ras2 activated [42] (349)	1 0.77	1 0.85	2 0.77	1 0.84	1 0.98	0	5 0.69	0	7 0.82	2 0.78	12 0.25	8 0.36	3 0.66	16 0.34	12 0.3	1 0.81	4 0.96	8 0.84
Tor1 activated [42] (59)	0	0	3 0.011	0	1 0.5	0	1 0.63	0	2 0.46	0	4 0.078	0	0	4 0.22	1 0.82	2 0.031	2 0.39	**7 1.9e-3**
UES genes [49] (17)	0	0	0	**2 3.3e-3**	0	**5 3.2e-8**	0	**3 9.5e-5**	2 0.072	1 0.13	0	0	**5 4.0e-7**	0	**4 1.2e-3**	0	0	0
Gis1 activated [49] (23)	0	0	1 0.17	**4 4.7e-6**	2 0.028	0	0	**7 1.7e-11**	1 0.46	1 0.17	1 0.47	0	**7 1.2e-9**	**5 2.0e-3**	1 0.49	0	1 0.41	1 0.51
Gis1 repressed [49] (25)	0	0	0	0	**11 8.5e-16**	**2 9.4e-3**	**4 6.8e-4**	0	0	**4 4.2e-5**	1 0.5	0	0	4 0.017	2 0.16	1 0.11	3 0.019	2 0.17

For each comparison the number of overlapping genes and a hypergeometrical p-value are shown. Entries that are significant at p<0.01 are shown in bold. The number of genes in each group is shown in parenthesis.

Several conclusions can be drawn from our results. First, repression mediated jointly by Gis1 and Rph1 in the presence of nutrients is just as important as is activation after nutrient depletion. Second, both Gis1 and Rph1 participate in gene activation after nutrient depletion. In fact, Rph1, which has not previously been implicated in this process, seems to be more important than Gis1 (Table 5). Third, since Gis1 and Rph1 have opposite effects on cluster S6, which overlaps with both the Tor1-activated and the Ras2-repressed genes, it is tempting to suggest that Gis1 and Rph1 could be differentially regulated by these two pathways in early stationary phase.

### Control of genes involved in glycerol metabolism by Gis1 and Rph1

Since Gis1 and Rph1 were found to jointly regulate glycerol accumulation after the diauxic shift (Figure 3) we next examined the genes involved in glycerol metabolism. We found that Gis1 and Rph1 regulate several of these genes, but their targets change with the growth phase. In log phase cells, Gis1 and Rph1 repress *HOR2* (3.0-fold, p = 8.8e-4) one of two genes encoding glycerol-3-phosphatase, the final enzyme in glycerol biosynthesis. However, they also repress *GCY1* (5.8-fold, p = 1.2e-9), which encodes an NADP-dependent glycerol dehydrogenase [55]. Thus, both production of glycerol and its further conversion to dihydroxyacetone is repressed by Gis1 and Rph1. The regulation is partly redundant, with at least *GCY1* being significantly derepressed also in the single mutants. The fact that both the synthesis and catabolism of glycerol is repressed by Gis1 and Rph1 may explain why no effect is seen on the log phase glycerol level in the mutants (Figure 3).

**Figure 5:**
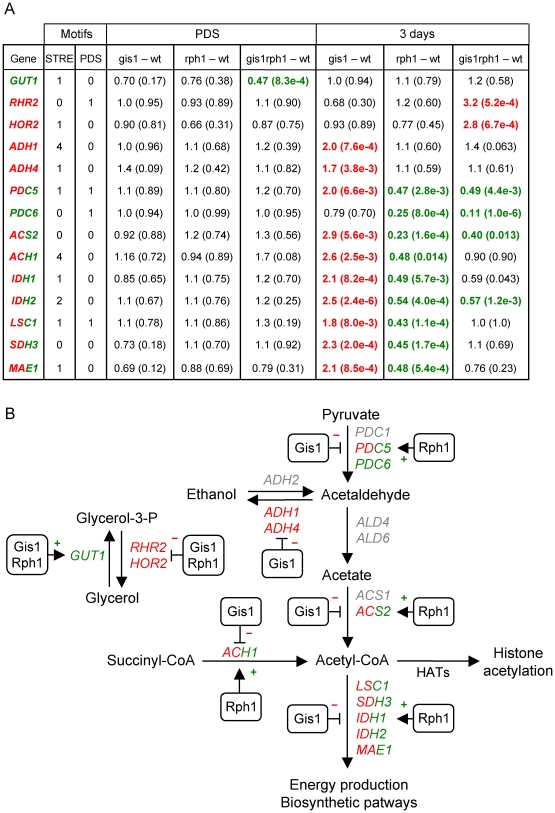
Effects of Gis1 and Rph1 on glycerol, acetate and acetyl-CoA metabolism. (A) Effects of *gis1* and *rph1* mutations on gene expression after the diauxic shift. The columns show fold changes and p-values for different genetic contrasts in PDS cells and after 3 days of culture. Genes that are significantly (p<0.02) downregulated in a mutant are shown in green and those that are upregulated in red. Genes are shown in red/green are upregulated in the *gis1* mutant and downregulated in the *rph1* mutant. Also shown are the number of STRE and PDS motifs in each promoter. (B) Glycerol and acetate metabolic pathways in yeast. The effects of Gis1 and Rph1 on genes encoding some key enzymes are shown. It should be noted that regulation in several cases is likely to be indirect, being mediated by the repression or activation of some other transcription factor.

In PDS cells, a significant effect is seen on *GUT1* encoding glycerol kinase, the first enzyme in glycerol degradation. *GUT1* is downregulated 2-fold in the *gis1 rph1* double mutant, indicating that it is redundantly activated by Gis1 and Rph1 (Figure 5). However, this was one of the few cases where a significant effect in the array data failed the 0.02 p-value test in the qPCR data (Table S1). After 3 days, *GUT1* is no longer affected, but the two glycerol-3-phosphatase genes are instead significantly upregulated in the *gis1 rph1* double mutant indicating that these two genes are repressed by Gis1 and Rph1 (Figure 5). Also in this case, the regulation is largely redundant, with no significant effects in the single mutants. We conclude that Gis1 and Rph1 prevent glycerol accumulation after the diauxic shift, first by activating its degradation, and later by repressing its synthesis. This is consistent with our finding in Figure 3 that the extracellular glycerol levels are significantly elevated (6.2-fold, p = 1.7e-3) after 2 days in the *gis1 rph1* double mutant, an effect that persists also after 3 and 4 days of culturing. Finally, we note that the single mutants had a much smaller effect on the glycerol level, which is consistent with the largely redundant gene regulation.

### Control of genes involved in acetate metabolism by Gis1 and Rph1

We proceeded to examine the genes involved in acetate metabolism. *ALD4* encodes the major aldehyde dehydrogenase that catalyses the last step in acetate formation. In log phase cells, *ALD4* is upregulated 3-fold (p = 1.5e-5) in the *gis1 rph1* double mutant, and a significant effect (2.4-fold, p = 1.9e-3) is also seen in the *rph1* single mutant. However, *ALD4* is no longer regulated by Gis1 or Rph1 after the diauxic shift, nor is *ALD6* encoding the other dehydrogenase involved in acetate production. Two other *ALD* genes are downregulated in the double mutant: *ALD2* (2.5-fold, p = 9.8e-8) and *ALD3* (2.9-fold, p = 7.0e-4). *ALD2* and *ALD3* are Msn2/4-dependent stress response genes [56], but they are not thought to function in acetate production. Instead, they are involved in the synthesis of β-alanine [57]. We conclude that the positive effect of Gis1 and Rph1 on acetate accumulation (Figure 3) must involve some other step than the conversion of acetaldehyde to acetate.

Indeed, we find that three adjacent metabolic steps are affected by Gis1 and Rph1 in stationary phase cells (Figure 5). Acetaldehyde can be formed either from pyruvate or ethanol. The latter reaction is reversible, but different isozymes catalyze the forward and reverse reactions. We find that the *PDC6* gene encoding a pyruvate decarboxylase is downregulated 9-fold in the double mutant and 4-fold in the *rph1* single mutant. *PDC6* is a minor isozyme, but it is strongly induced by nutrient stress, in particular sulfur limitation [58]. The *PDC5* gene, which encodes a second minor isozyme, is downregulated 4-fold in the *rph1* mutant but upregulated 2-fold in the *gis1* mutant. The double mutant shows a smaller but still significant downregulation. Gis1 and Rph1 thus have opposite effects on *PDC5* expression, with an *rph1* mutation being epistatic over a *gis1* mutation. In conclusion, Rph1 is expected to stimulate acetaldehyde formation by activating *PDC5* and *PDC6*, which in turn will result in more acetate being formed. A reduced conversion of pyruvate to acetaldehyde could thus explain why acetate levels are reduced in the *rph1* mutant (Figure 3).

Gis1 is also predicted to promote acetate formation, but by a different mechanism. Thus, the *gis1* mutant shows a significant upregulation of the two alcohol dehydrogenase genes *ADH1* and *ADH4* after 3 days (Figure 5). No effect was seen in the *rph1* or the double mutant. Adh1 and Adh4 are responsible for reduction of acetaldehyde to ethanol. They are therefore repressed after the diauxic shift, when yeast cells metabolize the ethanol that was previously formed. Our results suggest that Gis1 contributes to this repression. As a consequence more acetaldehyde will be converted into acetate in the *gis1* mutant, which is consistent with the reduced acetate levels in this mutant (Figure 3).

### Control of genes involved in acetyl-CoA metabolism by Gis1 and Rph1

Interestingly, the *ACS2* gene, which encodes a nuclear acetyl-CoA synthetase, is regulated in opposite ways by Gis1 and Rph1, similar to *PDC5* (Figure 5), though only its repression by Gis1 could be validated at p<0.02 by qPCR (Table S1). Acs2 is needed for histone acetylation [59], and an *acs2* mutant has an aging phenotype similar to that of a *sir2* mutant, including loss of silencing of rDNA and accumulation of extrachromosomal circles [60]. Another gene that contributes to acetyl-CoA formation is *ACH1*, encoding an acetyl-CoA transferase, and it is regulated in the same way (Figure 5). This prompted us to examine other genes involved in acetyl-CoA metabolism. We note that a set of 395 genes that are annotated with the Gene Ontology term *carboxylic acid metabolic process* is enriched in cluster S6, the Gis1-repressed Rph1-activated cluster that includes *ACS2* and *ACH1* (Table 3). This GO set comprises genes encoding TCA cycle enzymes and other enzymes that affect acetyl-CoA metabolism. When we examined the effects of *gis1* and *rph1* on this set of genes we found a highly significant pattern similar to that of *ACS2* and *ACH1* (Table 5). Thus, in stationary phase cells, these genes are upregulated in the *gis1* mutant (p = 3.0e-14), and downregulated in the *rph1* mutant (p = 4.2e-26). To refine the analysis, we used the KEGG pathway database [61] to identify enzymatic reactions involving acetyl-CoA, and examined the corresponding genes. This smaller set of genes (32 in total) shows the same pattern (Table 5). We conclude that genes involved in acetyl-CoA metabolism are, as a group, repressed by Gis1 and activated by Rph1 after 3 days.

### Promoters that are regulated by Gis1 and/or Rph1 are enriched for STRE and PDS motifs

To find out more about how Gis1 and Rph1 regulate different genes, we tested a list of known yeast regulatory motifs [62] for enrichment in the promoters of the genes that respond to a deletion of Gis1 and/or Rph1. We also carried out a *de novo* search for enriched motifs using BioProspector [63]. Several motifs were enriched in the promoters of these genes using one or both methods. They include the Xbp1 binding site discussed above [53], the Mig1 binding site [64], and the PAC [65], rRPE [66] and Rap1 [67] motifs, which are involved in ribosomal biogenesis. However, the two most frequent motifs were the STRE and PDS motifs. This is consistent with studies that found STRE and PDS motifs in several genes that are regulated by Gis1 and Rph1 [12], [21], [23], [47]. It is also consistent with data which show that Gis1 can bind to the PDS motif [24] and that Rph1 can bind to the STRE motif [23], [25]–[27].

An interesting new result, however, is that the STRE and PDS motifs appear to have different roles at different time points. Thus, the STRE motif is enriched in genes that are up-regulated during the log phase in response to deletions of Gis1 and/or Rph1 (Table 7). This suggests that Gis1 and Rph1 mainly repress genes in the log phase, acting through the STRE motif. The genes in this group are enriched for the gene ontology terms *glycogen metabolic process* and *response to oxidative stress*. After the diauxic shift, the STRE and PDS motifs are instead enriched in the promoters of genes that are down-regulated in response to deletions of Gis1 and Rph1 (Table 7). This suggests a role for Gis1 and Rph1 in activation through the STRE and PDS motifs upon glucose depletion. This is in agreement with findings that Gis1 plays a role in the induction of certain genes, such as *SSA3* and *GRE1*, during the diauxic shift [12]. However, the fact that Rph1 also plays an activating role after the diauxic shift has not previously been reported. Interestingly, even though the log phase repressed and PDS phase activated genes both have PDS and/or STRE motifs in their promoters, there is only a small overlap (31 genes) between the two groups of genes. It is thus not the same genes that are repressed in log phase and activated after the diauxic shift.

**Table 7:** Enrichment of STRE and PDS motifs in differentially expressed promoters.

Differential expression	STRE per promoter	STRE p-value	PDS per promoter	PDS p-value
up in [gis-wt] log	0.94	1.19e-01	0.50	1.16e-01
up in [rph1-wt] log	**1.44**	**5.12e-05**	0.25	4.19e-01
up in [gis1rph1-gis1] log	**1.14**	**1.95e-08**	0.23	9.42e-02
up in [gis1rph1-rph1] log	**2.00**	**3.76e-05**	0.17	9.22e-01
up in [gis1rph1-wt] log	**1.67**	**1.14e-15**	**0.34**	**2.95e-03**
down in [gis1-wt] PDS	**1.22**	**1.01e-04**	**0.41**	**2.63e-02**
down in [rph1-wt] PDS	**0.90**	**3.05e-02**	0.17	9.36e-01
down in [gis1rph1-gis1] PDS	**1.02**	**1.39e-08**	0.20	2.60e-01
down in [gis1rph1-rph1] PDS	**1.29**	**1.33e-05**	**0.54**	**5.56e-04**
down in [gis1rph1-wt] PDS	**0.94**	**5.37e-08**	**0.36**	**6.85e-06**
down in [gis1-wt] 3d	**1.00**	**1.60e-04**	**0.50**	**2.29e-04**
down in [rph1-wt] 3d	0.47	9.74e-01	0.12	6.94e-02
down in [gis1rph1-gis1] 3d	**0.60**	**1.28e-02**	0.16	9.30e-01
down in [gis1rph1-rph1] 3d	**0.96**	**1.00e-04**	**0.46**	**5.11e-04**
down in [gis1rph1-wt] 3d	**0.73**	**1.25e-03**	**0.27**	**2.80e-02**

For each group of differentially expressed genes, the average number of STRE and PDS motifs per promoter, along with p-values for the enrichment of STRE and PDS motifs is given. For comparison, among all yeast promoters, there is on average 0.47 STRE motifs and 0.15 PDS motifs per promoter. Enrichments that are significant at p<0.05 are shown in bold face.

### The preferred orientations of the STRE and PDS motifs differ between activated and repressed genes

There are several possible explanations why Gis1 and Rph1 could work through the same sequence motifs but produce different results (repression in log phase and activation after the diauxic shift) for different genes. One is that Gis1 and Rph1 recognize STRE and PDS motifs in different orientations or distances from the TATA box in different growth phases. Such effects have been reported for Rap1, which regulates ribosomal genes [68], [69], and a study of known transcription factor binding motifs in yeast suggested that it might be a more widespread phenomenon [70]. To test for such effects, we compared the positions and orientations of the STRE and PDS motifs in genes that are regulated by Gis1 and/or Rph1, to the positions and orientations of the motifs in all yeast promoters. Interestingly, we found a significant Rph1-dependent bias in the orientation of the STRE motif during both repression and activation (Table 8). Thus, genes that are repressed by Rph1 in the log phase prefer the forward orientation (AGGGG), with a p-value of 4.5e-3. Genes that are activated by Rph1 at the diauxic shift instead prefer the reverse orientation (CCCCT), with a p-value of 1.5e-2. For the PDS motif, we saw a bias for the forward orientation (AGGGAT) in genes that are activated by Gis1 at the diauxic shift and after 3 days, with p-values of 3.5e-2 and 1.1e-2, respectively (Table 8). These results show that the orientations of Gis1/Rph1 binding sites may influence how genes are regulated. In contrast, no clear biases were found for the positions of the STRE or PDS motifs within the promoters (data not shown).

**Table 8:** Average orientations of STRE and PDS motifs in the promoters of different sets of genes.

Genes considered	Repressed in log phase by Gis1^a^	Repressed in log phase by Rph1^b^	Activated in PDS by Gis1^c^	Activated in PDS by Rph1^d^	Activated at 3 d by Gis1^c^	Activated at 3 d by Rph1^d^	All ORFs
Number of promoters	44	146	59	173	115	369	5550
forward STRE/prom	0.80	0.64	0.49	0.40	0.41	0.32	0.23
reverse STRE/prom	0.70	0.47	0.71	0.61	0.5	0.34	0.25
total STRE/prom	1.50	1.11	1.20	1.0	0.91	0.67	0.48
forw/rev STRE	1.13	**1.38**	0.69	**0.66**	0.81	0.94	0.92
p-value bias STRE	0.23	**4.5e-3**	0.14	**1.5e-2**	0.29	0.60	
forward PDS/prom	0.18	0.13	0.31	0.11	0.27	0.12	0.080
reverse PDS/prom	0.11	0.11	0.14	0.087	0.13	0.089	0.081
total PDS/prom	0.30	0.25	0.44	0.20	0.40	0.21	0.16
forw/rev PDS	1.6	1.12	**2.25**	1.33	**2.07**	1.3	0.99
p-value bias PDS	0.29	0.43	**3.5e-2**	0.24	**1.1e-2**	0.14	

For each group of genes, the average number of forward and reverse oriented STRE and PDS motifs per promoter is shown, as well as the ratio between forward and reverse oriented motifs, with p-values for the deviation from the ratio in all ORFs. Deviations that are significant at p<0.05 are shown in bold face.

a Up in gis1-wt or gis1rph1-rph1.

b Up in rph1-wt or gis1rph1-gis1.

c Down in gis1-wt or gis1rph1-rph1.

d Down in rph1-wt or gis1rph1-gis1.

### A linear model of the roles of the STRE and PDS motifs in gene regulation by Gis1 and Rph1

To further assess the roles of the STRE and PDS motifs in regulation by Gis1 and Rph1, we used a linear model that was fitted to our data in such a way that differences in expression are explained by the presence or absence of STRE and PDS elements in different orientations (Table 9). In the log phase, the forward STRE motif is strongly associated with repression by both Gis1 and Rph1. The reverse orientation is also strongly associated with repression: the effects are smaller, but still highly significant. The forward PDS motif is also associated with repression by both Gis1 and Rph1, but not as strongly as the STRE motif. In contrast, the reverse PDS motif does not seem to have any significant effects. In conclusion, the STRE and PDS motifs are both associated with repression by Gis1 and Rph1 in the log phase, and both show a bias for the forward orientation.

**Table 9:** Linear model of the effects of STRE and PDS motifs in different orientations on Gis1/Rph1-dependent gene expression.

[Genetic contrast] and Growth phase	Forward STRE (AGGGG)	Reverse STRE (CCCCT)	Forward PDS (AGGGAT)	Reverse PDS (ATCCCT)
[gis1-wt] log	**0.16/9.5e-17**	**0.13/4.6e-10**	**0.15/3.2e-6**	0.092/4.8e-3
[rph1-wt] log	**0.31/4.0e-73**	**0.20/1.3e-29**	**0.13/6.6e-7**	0.064/1.9e-2
[gis1rph1-gis1] log	**0.45/1.3e-60**	**0.17/6.7e-10**	**0.16/2.5e-4**	0.086/4.0e-2
[gis1rph1-rph1] log	**0.37/2.7e-64**	**0.13/3.0e-8**	**0.15/1.6e-5**	−0.021/5.4e-1
[gis1rph1-wt] log	**0.7/1.1e-147**	**0.37/4.9e-38**	**0.25/8.7e-10**	0.036/3.8e-1
[gis1-wt] PDS	0.047/3.3e-1	−0.0018/7.6e-1	**−0.54/9.1e-50**	**−0.19/2.3e-7**
[rph1-wt] PDS	**−0.10/3.3e-11**	**−0.23/8.9e-35**	−0.076/5.7e-3	−0.067/1.9e-2
[gis1rph1-gis1] PDS	**−0.28/3.4e-27**	**−0.30/7.2e-29**	−0.11/8.2e-3	−0.11/1.3e-2
[gis1rph1-rph1] PDS	**−0.12/2.0e-9**	**−0.11/1.26e-5**	**−0.86/7.5e-111**	**−0.52/7.57e-37**
[gis1rph1-wt] PDS	**−0.22/1.2e-15**	**−0.32/2.0e-22**	**−0.74/2.8e-55**	**−0.44/1.2e-17**
[gis1-wt] 3 d	−0.048/8.8e-3	0.0021/7.5e-1	**−0.88/6.5e-76**	−0.11/2.5e-2
[rph1-wt] 3 d	−0.021/5.7e-1	−0.0016/1.0e-0	0.14/1.8e-3	0.068/1.3e-1
[gis1rph1-gis1] 3 d	**−0.20/3.7e-7**	**−0.19/2.6e-6**	0.20/1.5e-3	0.0039/9.5e-1
[gis1rph1-rph1] 3 d	**−0.33/1.1e-29**	**−0.16/2.2e-7**	**−1.0/3.4e-96**	**−0.19/1.7e-4**
[gis1rph1-wt] 3 d	**−0.31/1.8e-21**	**−0.17/3.6e-6**	**−0.75/1.23e-45**	−0.091/1.1e-1

For each contrast, the effect of each orientation of the STRE and PDS motifs on gene expression (log ratios) in different contrasts are shown, along with ANOVA p-values. Statistically significant values (at the 0.001 level) are highlighted in bold face.

After the diauxic shift, we see a very different pattern (Table 9). The STRE motif is now strongly associated with activation, mainly by Rph1. Interestingly, this activation also shows orientation bias, but now for the reverse orientation of the motif. The PDS motif is also strongly associated with activation after the diauxic shift, but unlike the STRE motif, this effect is strictly Gis1-dependent. There is also an orientation bias, but in this case the same orientation (forward) is favored as in log phase repression. In conclusion, the STRE and PDS motifs are both associated with activation after the diauxic shift. For the STRE motif, this effect is mostly dependent on Rph1 whereas activation by the PDS motif is strictly dependent on Gis1. Both effects show orientation bias. After 3 days, both motifs are still associated with activation, but the effects of the STRE motif are significant only in contrasts involving the double mutant. The orientation bias of the PDS motif is now even more pronounced.

**Figure 6:**
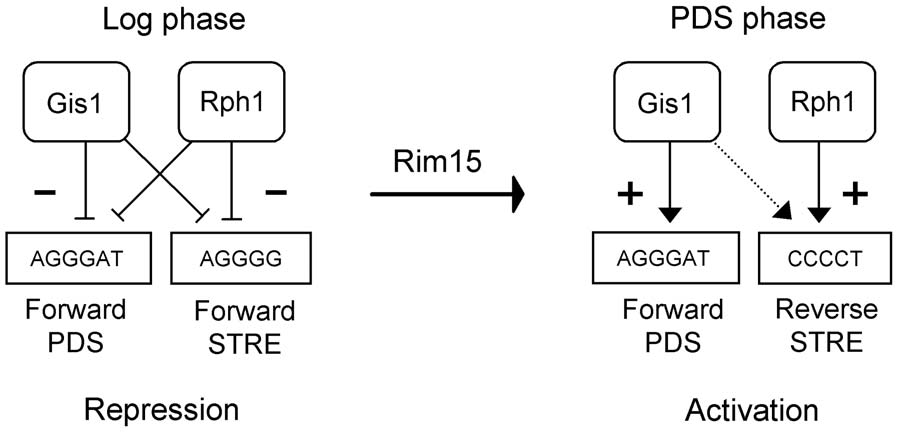
Model for gene regulation by Gis1 and Rph1 in different growth phases. Activation of transcription acting through a STRE or PDS motif is shown as an arrow, and repression as a cross-bar. A dashed line indicates a minor effect of Gis1 on STRE-mediated activation after the diauxic shift, which is only visible in an *rph1* mutant background.

Our interpretation of the results is shown in Figure 6. Gis1 and Rph1 are both associated with repression in the log phase, acting through the STRE and PDS motifs. The forward orientation of both motifs is favored. After the diauxic shift, Gis1 and Rph1 are both associated with activation. Activation through the STRE motif is largely dependent on Rph1, and favors the reverse motif. Activation through the PDS motif is strictly dependent on Gis1, and still favors the forward motif. These results are consistent with those in Table 8, where log phase repression shows a bias for the forward STRE motif, and PDS phase activation a bias for the reverse STRE motif and the forward PDS motif. We further note that genes that are repressed in the log phase by Gis1 also have an excess of forward PDS motifs in Table 8, which is consistent with the model, but the small number of genes made this effect non-significant. Finally, it should be noted that individual genes may differ from the model, since it reflects the average behavior of all genes regulated by Gis1 and/or Rph1.

## Discussion

While Gis1 has a well-established role in nutrient signaling, growth phase dependent gene regulation, and chronological aging [12], [14], [32], [47], nothing was previously known about the role, if any, of Rph1 in these processes. Gis1 and Rph1 have nearly identical zinc fingers and are thought to bind to similar DNA motifs. One would therefore expect to see a functional overlap between the two proteins. Gis1 and Rph1 do function redundantly as repressors of some genes, such as *PHR1* and *DPP1* [23], [24]. However, there is no previous evidence that Rph1 functions together with Gis1 in the PDS response. On the contrary, Rph1 has no effect on the PDS-driven induction of the *SSA3* gene, which is strictly dependent on Gis1 [21]. Nevertheless, the similarity of the two proteins, and the fact that Gis1 and Rph1 both are expressed after the diauxic shift [50], made us consider the possibility that Rph1 also could be involved in growth phase-dependent gene regulation. To test this hypothesis, we used microarrays to study gene expression in four yeast strains: a wild type, *gis1* and *rph1* single mutants, and a *gis1 rph1* double mutant. Gene expression was monitored at three different points: in the log phase, after the diauxic shift, and after 3 days. The 3 days point, here referred to as early stationary phase, was chosen since all glucose and ethanol has been consumed at this point (Figure 3). Density fractionations of yeast stationary phase cells have shown that after day 2, the cultures contain a mixture of quiescent cells, generated as daughter cells in the last cell division after the diauxic shift, and non-quisecent cells, which ultimately lose the ability to reproduce and become necrotic or apoptotic [71]. The cell population after 3 days is thus heterogenous, which should be kept in mind when interpreting the data.

### Rph1 and Gis1 are both involved in growth phase-dependent gene expression

Our results (Figure 4, Tables 1–3) show that Rph1 is indeed involved in growth phase-dependent gene regulation, and further reveal that there are many previously unknown targets for Gis1, not only during the PDS phase, but also in the log phase and early stationary phase. In fact, Gis1 and Rph1 regulate a large number of genes in all three growth phases, but in quite different ways. In the log phase, many genes are redundantly repressed by Gis1 and Rph1, but there are also examples of other modes of regulation (Table 1). After the diauxic shift, there is more variety (Tables 2 and 3). Some genes are repressed and some activated, either by Gis1 alone, Rph1 alone, or by both proteins. When Gis1 and Rph1 control the same gene they usually function synergistically, though some genes show redundant activation or repression.

We further found that a deletion of Rph1 resembles a deletion of Gis1 in that it has a clear effect on the expression of known targets of the TOR, PKA and Sch9 pathways (Table 5). Moreover, the predominant activity of Rph1 switches from repression in the log phase to activation after the diauxic shift, similar to Gis1. This switch is likely to be controlled by Rim15, a key regulator of growth phase dependent gene expression [10]–[13]. Consistent with this, we found that genes that are subject to Rim15-dependent activation after glucose depletion [42] are repressed by Rph1 in the log phase and activated by Rph1 after the diauxic shift (Table 5). We conclude that not only do the targets of Rph1 and Gis1 overlap, but the Rph1-regulated genes respond to the same signaling pathways as the Gis1-regulated genes. This does not mean, however, that the two proteins have identical functions. As noted above, there are many differences in how they control gene expression. One key difference seems to be that post-diauxic activation through the STRE motif mostly involves Rph1, whereas activation through the PDS motif is strictly dependent on Gis1 (Table 9). This is consistent with the known role of Gis1 as an activator of several PDS-driven genes [21].

### Dual roles of Gis1 and Rph1 in activation and repression

Our finding that Gis1 can act both as a repressor in the presence of glucose (in log phase) and as an activator in the absence of glucose (after the diauxic shift) is interesting in view of the fact that Gis1 is constitutively present in the nucleus. The localization of Gis1 is thus not regulated by Rim15 [12], even though genetic evidence suggests that the activity of Gis1 is controlled by Rim15. How Rim15 controls Gis1 function is not known, but we note that Rim15 accumulates in the nucleus under glucose limitation [11]. The fact that Gis1 seems to function mainly as an activator after glucose depletion, when Rim15 is present in the nucleus, therefore suggests the possibility that Rim15-dependent phosphorylation could transform Gis1 from a repressor to an activator (Figure 6). A similar argument can be made for Rph1, which also seems to be regulated by Rim15. In this context we note that the kinase activity of Rim15 controls a limited proteolysis of Gis1 [72]. It is possible that the switch between the activating and repressing activities of Gis1 could involve such limited proteolysis.

Such a model would not be unprecedented. There are several examples of transcription factors that can switch function [73]. We further note that our model provides a possible explanation for the dual role of Sch9 in STRE-dependent gene regulation. Thus, Sch9 activates expression of genes containing STRE motifs in the presence of Rim15, and represses their expression in its absence [12]. It is possible that this effect is mediated by Gis1 (and also Rph1) acting downstream of Sch9, and that the Rim15-dependent switch between activation and repression of the target genes reflects a switch between activator and repressor roles for Gis1 and Rph1.

The promoters of genes that are regulated by Gis1 and Rph1 are enriched for PDS and STRE motifs, which suggests a direct mode of regulation for many of these genes. Other genes that lack STRE and PDS motifs are likely to be indirectly regulated by Gis1 and Rph1, through repression or activation of some other transcription factor. One example of this may be cluster S6 (Table 3), where some genes are likely to be regulated by Xbp1, which is repressed by Gis1 and Rph1 in the preceding log phase (Table 1). There are probably many cases of indirect regulation that remain to be identified. Of the 1521 genes in Figure 4, more than half (810) have neither STRE motifs nor PDS motifs in their promoters. In particular, it is likely that when Gis1 and Rph1 seem to act as repressors after the diauxic shift, this may be due to indirect effects, *i.e.* activation of a repressor. Consistent with this, cluster P5, which is repressed by Gis1 and Rph1 in the PDS phase, is not enriched for either STRE or PDS motifs, and cluster S5, which is repressed by Gis1 and Rph1 after three days, is only weakly enriched (p = 0.04) for STRE motifs.

### Orientation bias of the STRE and PDS motifs

Interestingly, we found that the preferred orientation of the STRE motif differs between log phase repressed and PDS phase activated genes (Tables 8 and 9). An orientation bias was also seen for the PDS motif, though the forward orientation is favored during both activation and repression in that case (Table 8). These surprising biases suggest that the abilities of Gis1 and Rph1 to activate or repress transcription depend at least in part on the promoter context. There are few examples of orientation bias for promoter motifs, but one example is Rap1, another yeast transcription factor that can function both as an activator and as a repressor [68], [69]. We note that since most of the STRE-associated activation is dependent on Rph1, the evidence for a switch in orientation bias is stronger for Rph1, but we cannot rule out that it also affects Gis1. How Rph1 (and perhaps also Gis1) distinguishes between the two orientations of the STRE motif remains to be determined. One possibility is that a Rim15-dependent signal determines which orientation of the motif is preferred, and thus also what target genes are regulated and whether the protein acts as a repressor or as an activator (Figure 6). In the case of the PDS motif, where the forward orientation always is favored, it appears that it is only the switch from repressor to activator function that is regulated. This mode of regulation may be specific for Gis1, since there is little evidence that Rph1 activates transcription through the PDS motif.

### Gis1 and Rph1 jointly regulate glycerol and acetate accumulation

The most striking phenotypic effect of the *gis1* and *rph1* deletions is to decrease acetate accumulation and increase glycerol accumulation after the diauxic shift (Figure 3). There is, however, an important difference between the two effects. The increase in extracellular glycerol is most clearly seen in the *gis1 rph1* double mutant, whereas the effect on acetate accumulation is pronounced also in the single mutants. This suggests that Gis1 and Rph1 act redundantly in promoting glycerol accumulation, but synergistically in reducing acetate accumulation. This is consistent with our finding that Gis1 and Rph1 redundantly regulate genes involved in glycerol metabolism (*GUT1*, *HOR2*, *RHR2*), but instead have distinct targets (*ADH1/ADH4* and *PDC6*, respectively) affecting acetate accumulation (Figure 5).

The fact that Gis1 and Rph1 control glycerol and acetate metabolism is interesting since *gis1* strains have an accelerated aging phenotype, and since glycerol and acetate accumulation both affect aging in yeast. However, the effects of Gis1 on glycerol and acetate metabolism are the opposite of what one would expect from the aging phenotype of *gis1* mutants. Glycerol accumulation is associated with an extended lifespan: *sch9* mutants live longer and accumulate more glycerol [14]. Loss of Gis1 suppresses the longevity phenotype of *sch9*, and shortens the lifespan [14]. One might therefore expect *gis1* to have the opposite effect of *sch9*, but the *gis1* mutant instead resembles the *sch9* mutant in that it accumulates more glycerol. Conversely, the short-lived *gis1* mutant accumulates less extracellular acetate (Figure 3), even though a short lifespan is associated with increased acetate accumulation [41].

### Acetate accumulation, acetyl-CoA metabolism and aging

These results raise the question whether the effects of acetate on chronological aging could be indirect, *i.e.* mediated by some other metabolite or process that depends on acetate. In support of this idea, we note that the effect of acetate on aging is delayed. Yeast cultures accumulate acetate in early stationary phase, but this acetate is rapidly consumed (Figure 3). It is only much later that the effect on aging is seen [39], [41]. This rules out an immediate effect of acetate on survival, and instead suggests that cells transiently exposed to acetate undergo a reprogramming that reduces long-term survival. This in turn suggests that epigenetic changes, such as histone modifications, could be involved. Histone acetylation promotes replicative aging in yeast [45], and Msn2/Msn4-dependent control of Sir2 activity plays a key role in this process [44]. Furthermore, an *acs2* mutant that lacks a nuclear acetyl-CoA synthetase needed for histone acetylation [59] has an accelerated aging phenotype similar to a *sir2* mutant [60]. It should be noted that this result, while implicating acetyl-CoA formation in replicative aging, was surprising since a delayed aging phenotype was expected [60]. The effect of *acs2* on chronological aging, and the effect of *ACS2* overexpression on either type of aging also remain to be investigated.

That being said, our finding that *ACS2* and several other genes involved in acetyl-CoA metabolism are repressed by Gis1 raises the possibility that the accelerated chronological aging of *gis1* cells and of cells exposed to acetate both could be mediated at least in part by acetyl-CoA. In cells exposed to acetate, uptake and conversion of acetate to acetyl-CoA could increase the nuclear acetyl-CoA level, with a resulting increase in histone acetylation and loss of silencing. Loss of silencing can cause replicative aging [44]–[46], but it could also be detrimental to survival of resting cells, thus contributing to chronological aging. The reduced acetate accumulation in the *gis1* mutant (Figure 3) could on the other hand be due to increased conversion of acetate to acetyl-CoA (Figure 5), which would explain why this mutant shows accelerated aging even though it accumulates less acetate.

## Materials and Methods

### Yeast strains and growth conditions

The BY4742 background (*MATα his3-Δ1 leu2-Δ0 lys2-Δ0 ura3-Δ0*) was used for all experiments. The *gis1* and *rph1* single deletion strains Y14031 and Y16165 were from the Euroscarf collection, while the *gis1 rph1* double deletion strain, H1437, was made by crosses of the appropriate single deletions followed by tetrad dissection. Yeast cells were grown in rich 2% glucose media (YPD). Overnight pre-cultures where diluted to an OD_600_ of 0.1, and then kept in continuous log phase by repeated dilutions during the next 24 hours in order to ensure that no stationary phase transcripts remained. After the final dilution to an OD_600_ of 0.1, log phase cells were harvested when the culture reached an OD_600_ of 0.4 (time point 0). Diauxic shift cells were harvested 9 h later at an OD_600_ of 10, when reverse transcriptase experiments revealed that the *SSA3* gene was induced, and early stationary phase cells three days later, at an OD_600_ of 18. For the reverse transcriptase experiments, cells were harvested at 5, 9, 13, 22, 30, 46 and 70 h after time point 0. The cells were pelleted by centrifugation for 5 min, and then immediately frozen in liquid nitrogen. For the microarray experiments, all samples were prepared in biological triplicates.

### Reverse transcriptase analysis of mRNA

RNA was prepared from yeast cells using the RiboPure™-Yeast kit (Ambion). Equal amounts of RNA from each sample were used as templates for reverse transcriptase with oligo dT primers, and the cDNA thus produced was used as template in a PCR with the following gene-specific oligonucleotide primers: *SSA3* forward, 5′-TTC TAT CAA CCC GGA TGA GG-3′, *SSA3* reverse, 5′-AAT TTG AGG CAC ACC TCT GG-3′, *ACT1* forward, 5′-CGT TCC AAT TTA CGC TGG TT-3′, *ACT1* reverse, 5′-CGG TGA TTT CCT TTT GCA TT-3′. The amplified DNA was separated on 1.5% agarose gels.

### Quantitative RT-PCR

Three µg of RNA from each array sample was used as template for cDNA synthesis using the iScript™ Advanced cDNA Synthesis Kit for RT-qPCR (Bio-Rad). The cDNA was diluted 10 times and then used for quantitative Real-Time PCR using SsoFast™ Eva Green Supermix (Bio-Rad) on a Bio-Rad CFX96™ RealTime System. The primers used are listed in Table S2. At least 3 technical replicates were run for each biological replicate. Fold changes were calculated using the delta–delta cycle threshold method and p-values using the same pipeline as for the microarray data. The *TDH3* gene encoding glyceraldehyde-3-phosphate dehydrogenase was used as a reference.

### HPLC analysis

For the HPLC analysis, yeast strains were grown in triplicate under the same conditions as in the microarray experiment. At the indicated time-points, cells were pelleted by centrifugation after which the supernatants were passed through a 0.2 µm sterile filter and frozen at −80°C. The samples were analyzed on a Rezex ROA-Organic Acid H+ (8%) column from Phenomex, using glucose, ethanol, acetate and glycerol standards to identify the corresponding peaks and calculate concentrations.

### Microarray experiments and data processing

RNA was prepared from frozen yeast cells using the RiboPure™-Yeast kit (Ambion). The purified RNA was hybridized to Affymetrix YG-S98 arrays using the standard Affymetrix protocol. The raw data was processed using the Affy package [74] from Bioconductor [75]. The GCRMA normalization pipeline [76] was used. Because overall expression levels might differ between the different time points, data from each time point was normalized and analyzed further separately. Five genotype contrasts from each of the three growth phases were considered: *gis1*–wt, *rph1*–wt, *gis1rph1*–*gis1*, *gis1rph1*–*rph1* and *gis1rph1*–wt (see Table 7 for a complete list of contrasts). For each contrast, genes were tested for differential expression with a moderated t-statistic, with FDR correction to compensate for multiple hypothesis testing [77], [78]. A gene was considered differentially expressed if its expression differed at least two-fold between the two conditions, and the FDR corrected p-value was below 0.01. This resulted in 30 lists of genes, with significantly up- and down-regulated genes for each contrast. The *GO term finder* tool from SGD [79] was used to test groups of genes for enrichment of Gene Ontology annotations, with a threshold on the p-value of 0.001. The expression profiles were also clustered in MEV [80], using average linkage clustering and the Pearson Correlation distance metric.

To determine the relative influence of Gis1 and Rph1 on the expression of a particular gene, the following measure was used: *Gis1/Rph1 influence ratio  =  abs(g) - abs(r)*. Here, *g* is the expression log ratio between the *gis1* strain and the wild type, and *r* is the expression log ratio between the *rph1* strain and the wild type. A value of 0 indicates equal influence of Gis1 and Rph1, positive values indicate more influence of Gis1, and negative values more influence of Rph1. For genes regulated by both Gis1 and Rph1 we wanted to estimate the degree of redundancy in their regulation. For this, we defined a redundancy coefficient as follows: *Redundancy  =  (d−(g+r))/d*. Here, *d* is the log ratio between the *gis1 rph1* strain and the wild type, and *g* and *r* the same as above. A redundancy coefficient of 0 is interpreted as no redundancy, whereas a value of 1 indicates complete redundancy. It should be noted, however, that values may also fall outside this range, for example negative values indicate that the single deletions have a bigger effect on gene expression than the double deletion. In the present paper, we refer to genes with a redundancy coefficient above 0.5 as being redundantly regulated by Gis1 and Rph1, and to genes with a redundancy coefficient below 0.5 as being synergistically regulated. The micro-array data is available from ArrayExpress [81] with accession number E-TABM-496.

### Promoter sequence analysis

Promoter sequences for each gene were extracted from RSAT [82], using the sequence from the start codon and 800 bp upstream, or until the next gene was reached. When searching for enriched motifs in the promoter sequences, we used a list of known yeast motifs [62], along with additional motifs such as the PDS motif. A t-test was used to test for enrichment of each motif in the promoters of differentially expressed genes, against a background of all other promoter sequences. To statistically test for differences in promoter orientation, a one-sided hyper-geometrical test was used. We also performed a *de novo* search for enriched motifs, using BioProspector [63].

Similar to Bussemaker et al. [83], we fitted a linear model to our data, in order to determine what influence the orientations of the STRE and PDS motifs have on gene expression in various contrasts. In fitting the model, genes were weighted on absolute log ratios, so that genes with bigger differences in expression were given higher weight. For each orientation of the STRE and PDS motifs we obtained a coefficient describing the effect of the motif and an ANOVA p-value.

## Supporting Information

Table S1 **qPCR validation of microarray data.** The columns show fold changes relative to the wild type with p-values in parentheses. Significant (p<0.02) changes are shown in bold. ^a^ *ADH1* was not included in the hierarchical clustering since its fold change in the *gis1Δ* mutant (1.98) fell just below our 2.00 threshold.
(PDF)

Table S2 **Primers used for qPCR.**
(PDF)
