# Supplementary material for: Gis1 and Rph1 Regulate Glycerol and Acetate Metabolism in Glucose Depleted Yeast Cells
Source: PLoS One. 2012 Feb 21;7(2):e31577. doi: 10.1371/journal.pone.0031577 (PMC3283669; doi:10.1371/journal.pone.0031577)
Supplement: Table S1 — qPCR validation of microarray data. The columns show fold changes relative to the wild type with p-values in parentheses. Significant (p<0.02) changes are shown in bold. a ADH1 was not included in the hierarchical clustering since its fold change in the gis1Δ mutant (1.98) fell just below our 2.00 threshold. (PDF) [file pone.0031577.s001.pdf]

Table S1. qPCR validation of microarray data

Time	Gene	Cluster	Cluster description	*gis1*Δ		*rph1*Δ		*gis1*Δ *rph1*Δ	
				Microarray	qPCR	Microarray	qPCR	Microarray	qPCR
LOG	*PHO11*	L2	Act. by Gis1 and Rph1	**0.53 (4.1e-4)**	**0.41 (3.0e-4)**	**0.65 (1.4e-2)**	**0.57 (1.7e-2)**	**0.1 (1.6e-10)**	**0.15 (6.9e-8)**
	HXT4	L4	Repression by Rph1	1.4 (1.0e-1)	2.0 (4.3e-2)	**2.5 (8.3e-3)**	2.2 (3.7e-2)	**4.5 (3.0e-6)**	**7.2 (1.7e-5)**
	HXT5	L6	Redundant repression	1.0 (9.2e-1)	1.2 (6.0e-1)	1.2 (7.3e-1)	1.2 (4.8e-1)	**17.6 (1.6e-15)**	**10.0 (4.1e-7)**
PDS	*SSA3*	P2	Activation by Gis1	**0.11 (8.3e-18)**	**0.14 (2.5e-8)**	0.88 (3.4e-1)	1.2 (3.7e-1)	**0.07 (1.8e-19)**	**0.09 (1.0e-9)**
	GRE1	P2	Activation by Gis1	**0.07 (1.2e-15)**	**0.07 (2.5e-8)**	0.78 (1.4e-1)	0.87 (5.0e-1)	**0.04 (6.2e-18)**	**0.03 (4.1e-10)**
	PHO89	P3	Act. by Gis1 and Rph1	**0.25 (9.6e-10)**	**0.26 (1.0e-6)**	**0.36 (2.4e-4)**	**0.45 (4.8e-4)**	**0.06 (1.3e-16)**	**0.06 (1.6e-10)**
	SPS100	P3	Act. by Gis1 and Rph1	**0.16 (8.8e-14)**	**0.11 (4.4e-8)**	**0.49 (1.0e-3)**	**0.30 (1.4e-4)**	**0.04 (4.0e-20)**	**0.03 (1.6e-10)**
	GUT1	P3	Act. by Gis1 and Rph1	0.7 (1.7e-1)	0.66 (5.7e-1)	0.76 (3.8e-1)	0.46 (3.2e-1)	**0.47 (8.3e-4)**	0.35 (1.0e-1)
3 DAYS	*SSA3*	S1	Activation by Gis1	**0.24 (4.1e-13)**	**0.16 (1.4e-5)**	0.97 (7.7e-1)	1.3 (4.4e-1)	**0.20 (2.9e-14)**	**0.12 (2.5e-6)**
	GRE1	S1	Activation by Gis1	**0.04 (5.6e-18)**	**0.01 (2.3e-10)**	1.1 (5.9e-1)	**3.4 (3.6e-3)**	**0.23 (1.2e-9)**	**0.14 (1.5e-5)**
	SPS100	S1	Activation by Gis1	**0.16 (7.1e-14)**	**0.16 (2.1e-5)**	1.0 (9.6e-1)	1.3 (4.4e-1)	**0.04 (3.3e-20)**	**0.06 (1.7e-7)**
	PDC6	S2	Activation by Rph1	0.79 (4.4e-1)	0.79 (5.3e-1)	**0.25 (8.0e-4)**	0.35 (2.4e-2)	**0.11 (3.0e-8)**	**0.27 (5.9e-3)**
	RHR2	S3	Unclear	0.68 (3.0e-1)	0.50 (2.4e-1)	1.2 (6.0e-1)	1.6 (4.9e-1)	**3.2 (5.2e-4)**	**6.2 (3.5e-3)**
	HOR2	S5	Rep. by Gis1 and Rph1	0.93 (8.9e-1)	0.90 (7.6-1)	0.77 (4.5e-1)	1.0 (1.0e-0)	**2.8 (6.7e-4)**	**5.5 (4.1e-4)**
	HXT2	S5	Rep. by Gis1 and Rph1	3.0 (2.4e-1)	1.8 (4.1e-1)	1.0 (9.5e-1)	1.0 (9.8e-1)	**19.9 (8.5e-5)**	**6.6 (1.3e-2)**
	ERG6	S6	Gis1 rep. Rph1 act.	**5.4 (6.7e-5)**	**5.3 (1.3e-3)**	**0.16 (2.3e-5)**	0.41 (7.6e-2)	1.4 (1.7e-1)	2.4 (5.7e-2)
	ACS2	S6	Gis1 rep. Rph1 act.	**2.9 (5.6e-3)**	**2.9 (1.2e-2)**	**0.23 (1.6e-4)**	0.35 (2.4e-2)	**0.40 (1.3e-2)**	1.1 (8.3e-1)
	ACH1	S6	Gis1 rep. Rph1 act.	**2.6 (2.5e-3)**	**3.4 (1.5e-3)**	**0.48 (1.4e-2)**	**0.28 (3.6e-3)**	0.90 (9.0e-1)	1.1 (8.5e-1)
	ADH1		Not included [a]	**2.0 (7.6e-4)**	**5.0 (1.1e-3)**	1.1 (6.0e-1)	1.9 (1.5e-1)	1.4 (6.3e-2)	2.4 (4.3e-2)
