# Supplementary material for: Gis1 and Rph1 Regulate Glycerol and Acetate Metabolism in Glucose Depleted Yeast Cells
Source: PLoS One. 2012 Feb 21;7(2):e31577. doi: 10.1371/journal.pone.0031577 (PMC3283669; doi:10.1371/journal.pone.0031577)
Supplement: Table S2 — Primers used for qPCR. (PDF) [file pone.0031577.s002.pdf]

Table S2. Primers used for qPCR

Primer	Sequence
TDH3 for	5´-GTTGACGGTCCATCCCACAA-3´
TDH3 rev	5´-CCATACCGGTCAACTTACCTTG-3´
PHO11 for	5´-GGTACGTTCCACAAGGTGCT-3´
PHO11 rev	5´-CCAGTCCCAGAAAAAGGTCA-3´
HXT4 for	5´-TGTTTTCGCTTCTGTTGGTG-3´
HXT4 rev	5´-TCAAGAAACCCCAGATCCAG-3´
HXT5 for	5´-AGTTTTGGCGCCTATGCTAA-3´
HXT5 rev	5´-ATGGTACCCTCCATTGGACA-3´
SSA3 for	5´-CAGGCTAAGAATCAGCTTGAATCG-3´
SSA3 rev	5´-TCAATGGTTTCCTGAGACGCTG-3´
GRE1 for	5´-CAGTTTGGCGGTAACGACTT-3´
GRE1 rev	5´-GCGGTTACTTTGAGCACCTC-3´
PHO89 for	5´-GGGGCAAACGACGTGGCGAA-3´
PHO89 rev	5´-GCCTGCCAAGACAGCACCCA-3´
SPS100 for	5´-TGTGAGCCAACTCTGATTGC-3´
SPS100 rev	5´-ATGCCACGGTACTGTTGTGA-3´
GUT1 for	5´-GTCAGAAGGTCTCCGACAGC-3´
GUT1 rev	5´-CACGGCAACTTCCCAATACT-3´
PDC6 for	5´-TCCCCTCTATCTCCGCTCAGGC-3´
PDC6 rev	5´-ACCAAATTCGCTGGCAACCCCA-3´
RHR2 for	5´-GGGTAGAAACGGTTTGGGTTTC-3´
RHR2 rev	5´-AACGATTTTACAGCCAGCAGCC-3´
HOR2 for	5´-AGGAATGGCTTAGGATATCC-3´
HOR2 rev	5´-CTTACAACCGGCGGCTTTTC-3´
HXT2 for	5´-TTGCCGAATCCTATCCTTTG-3´
HXT2 rev	5´-ACCAAACAGCCCATGAAGAC-3´
ERG6 for	5´-CAACTCTGCCCAAAAGGAAG-3´
ERG6 rev	5´-CTATCGAGGCAGCGAAACTC-3´
ACS2 for	5´-AAACAACGCTACTGAAGGTGATGC-3´
ACS2 rev	5´-TTTGGTGAGGCGAAAGGACC-3´
ADH1 for	5´-GGTGCTGTTCTAAAGGCCACTG-3´
ADH1 rev	5´-GCATACCGACCAAAACGGTG-3´
ACH1 for	5´-ACACGGCTACGCCTTCGTTCG-3´
ACH1 rev	5´-GGGCGTATTTGGTGGGACCTGG-3´
